# Termination-Accelerated Electrochemical Nitrogen Fixation on Single-Atom Catalysts Supported by MXenes

**DOI:** 10.1021/acs.jpclett.2c00195

**Published:** 2022-03-23

**Authors:** Kaifeng Niu, Lifeng Chi, Johanna Rosen, Jonas Björk

**Affiliations:** † Department of Physics, Chemistry and Biology, IFM, Linköping University, 581 83 Linköping, Sweden; ‡ Institute of Functional Nano & Soft Materials (FUNSOM) and Jiangsu Key Laboratory for Carbon-Based Functional Materials & Devices, Soochow University, Suzhou 215123, P. R. China

## Abstract

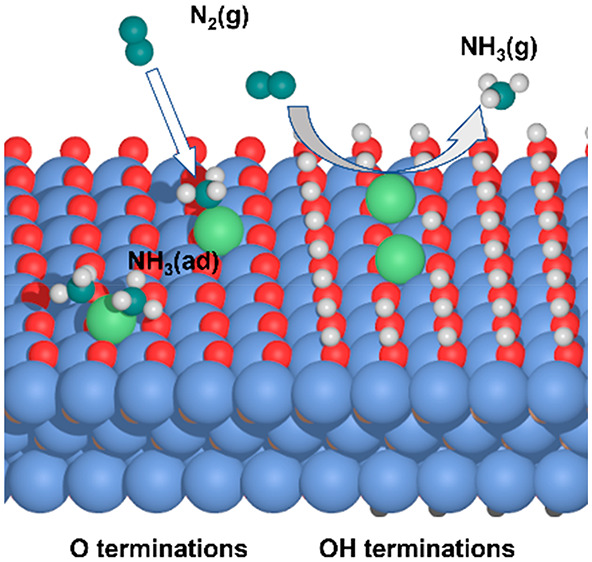

The synthesis of ammonia (NH_3_) from nitrogen (N_2_) under ambient conditions is of great significance but hindered by the lack of highly efficient catalysts. By performing first-principles calculations, we have investigated the feasibility for employing a transition metal (TM) atom, supported on Ti_3_C_2_T_2_ MXene with O/OH terminations, as a single-atom catalyst (SAC) for electrochemical nitrogen reduction. The potential catalytic performance of TM single atoms is evaluated by their adsorption behavior on the MXene, together with their ability to bind N_2_ and to desorb NH_3_ molecules. Of importance, the OH terminations on Ti_3_C_2_T_2_ MXene can effectively enhance the N_2_ adsorption and decrease the NH_3_ adsorption for single atoms. Based on proposed criteria for promising SACs, our calculations further demonstrate that the Ni/Ti_3_C_2_O_0.19_(OH)_1.81_ exhibits reasonable thermodynamics and kinetics toward electrochemical nitrogen reduction.

Ammonia synthesis, based on the Haber–Bosch process (N_2_ + H_2_ → NH_3_), has been considered as one of the most important industrial catalytic reactions and has had a critical role in the growth of human population for over 100 years.^1,2^ Nevertheless, harsh conditions including high pressure (200–400 atm) and moderately high temperature (400–600 °C) are required, which contributes not only to the large consumption of global energy but also to the emission of greenhouse gas.^3−5^ Alternatively, the electrochemical nitrogen reduction reaction (NRR) has emerged as a sustainable strategy in which NH_3_ can be synthesized by using H_2_O and N_2_ as raw materials on the cathode under ambient conditions.^6,7^ Tremendous efforts have been devoted into the rational design of effective catalysts toward NRR, including noble metals,^8^ non-transition metals,^9^ carbon nanotubes,^10^ and conducting polymers.^11^ However, the practical challenge remains at the slow reaction kinetics resulting from the nonpolar N≡N bonds, leading to limited ammonia selectivity and unsatisfactory yield.^12^

Single-atom catalysts (SACs), which contain isolated and exclusively dispersed transitional metal atoms, have been put forward as a promising class for different catalytic systems including NRR with high atomic utilization.^13,14^ For instance, single Fe atoms anchored in N-doped porous carbon are superior to commercial Pt/C catalysts for electrochemical oxygen reduction.^15^ In particular, the undercoordinated atomic environment of single atoms due to the active d-electrons would effectively enhance the adsorption and activation of N_2_ in NRR.^16^ As a result, the SACs can achieve outstanding catalytic performance for the production of ammonia compared to their bulk counterparts in terms of both activity and selectivity.^7,17^ For example, Li and co-workers have successfully synthesized Ru single atoms supported on Cu oxides (Ru/Cu_*x*_O_*y*_), which exhibit outstanding catalytic performance with an NH_3_ yield rate of 42.4 μg/(mg_cat._ h) and a Faradaic efficiency up to 14.1%.^18^ Nevertheless, the experimental discovery of SACs toward highly efficient NRR is mostly based on a trial-and-error approach, consuming much time, expense, and manpower.

Considering that SACs contain isolated metal atoms on the support materials, a central factor for fabricating stable SACs is to create a strong interaction between the single atom and the substrate.^19,20^ In this regard, density functional theory (DFT) calculations can be used as an effective approach for the fast screening of potential SACs and providing guidelines to design effective catalytic systems.^21,22^ For example, Reuter et al. have generated a database for 27 transition metal atoms on the vanadium disulfide (VS_2_) substrate and predicted that Ta would exhibit high activity toward electrochemical NRR.^23^ Similarly, Yang and co-workers have examined the feasibility for anchoring single transition metal atoms on the MoS_2_ monolayer. By calculating the binding energy of metal atoms on the MoS_2_ and the adsorption energy of N_2_ on the metal active sites, the Mo single atom is expected to have the best catalytic performance.^24^ Nevertheless, the agglomeration of isolated single metal atoms to nanoparticles is usually inevitable, leading to a decrease in the catalytic activity.^25^ Accordingly, employing appropriate materials for anchoring single atoms plays a vital role in promoting the catalytic performance. Previous studies have shown that two-dimensional (2D) materials such as C_2_N monolayers and nitrogen-doped porous carbon can be utilized as substrates for embedding transition metal atoms and improving the catalytic performance.^26,27^ Of importance, MXenes, as a large family of 2D layered materials with the general formula of M_*n*+1_X_*n*_T_*z*_ (M, X, and T refer to transition metals, C/N, and termination groups, respectively),^28^ exhibit great potential for electrocatalysis applications due to their high thermal stability and tunable electronic properties.^29^ Previous theoretical investigations have predicted that MXenes containing Ti and/or Mo atoms may possess high catalytic activity toward electrochemical NRR.^30−32^ Moreover, tuning surface chemical states of basal planes of MXenes has been proved as an effective approach to increase the catalytic activity. As an example, Zhi and co-workers have shown Fe-modified Ti_3_C_2_T_*z*_ MXene nanosheets present excellent Faradaic efficiency, outperforming other MXene-based NRR catalysts.^33^ In addition, the MXene structure provides adequate adsorption sites for anchoring single atoms because of their high degree of freedom including termination species and their distribution. Very recently, Ge et al. have theoretically proposed that single Ir atom decorated *v*-Mo_2_CT_*z*_ MXene (*v* represents the termination vacancy) possesses the optimal catalytic performance toward electrochemical NRR. The high catalytic activity is ascribed to synergetic effects of Ir atom and O terminations.^34^ Despite that great success has been achieved in SACs/MXenes toward electrochemical NRR, most studies have focused on bare MXenes and/or O terminated MXenes.^35,36^ Taking into account that MXenes with O terminations in solution unavoidably form other terminations, such as OH groups, leads to uncertainty of the catalytic performance and stability.^37,38^ In addition, studies focusing on the catalytic performance of single atoms supported on MXenes with a mixture of termination groups are limited in number. Therefore, it is necessary to take the OH terminations into consideration for both the stabilization of single metal atoms and the influence on the catalytic performance.

In the present work, we have investigated the feasibility for anchoring single transition metal atoms on the Ti_3_C_2_T_*z*_ MXene with different terminations and their catalytic performance toward NRR by first-principles calculations. In all, atoms of 19 different transition metals (TMs), including Sc, Ti, V, Cr, Mn, Fe, Co, Ni, Cu, Zn, Zr, Nb, Mo, Tc, Ru, Rh, Pd, Ag, and Cd, are examined on fully O-terminated and O/OH-terminated Ti_3_C_2_ MXene. The promising SACs are screened based on three criteria: (i) the stability for anchoring transition metal atoms to achieve single-atom dispersion, (ii) the adsorption of N_2_ being sufficiently strong to proceed hydrogenations, and (iii) the adsorption of NH_3_ being relatively weak so that active sites are available in further reduction cycles. Despite that the O terminations provide stable adsorption for single transition metal atoms, agglomeration is shown to be an issue for the fabrication of stable SACs in practical applications.^39,40^ By comparison of the adsorption of single atoms and their corresponding dimer structure, a selection of transition metals such as Sc, Nb, Cu, Cr and Ni are expected to form SACs with high stability. Although the N_2_ can be stabilized on most of the single transition metal atoms, further calculations suggest that SACs on Ti_3_C_2_O_2_ would suffer from the strong NH_3_ adsorption, resulting in a limited life cycle. Nevertheless, introducing OH terminations can both effectively reduce the adsorption of single NH_3_ and suppress the coadsorption of two NH_3_ molecules. As a result, the Ni/Ti_3_C_2_O_0.19_(OH)_1.81_ (labeled as Ti_3_C_2_T_2_ MXenes for convenience) is predicted to possess the best catalytic performance. DFT calculations show that OH terminations will not strongly influence the hydrogenation steps while the desorption of NH_3_ molecules is promoted. In addition, thermodynamics and kinetic analysis suggest that Ni/Ti_3_C_2_T_2_ can be used as an effective catalyst toward electrochemical NRR.

Our first criterion for employing SACs into electrochemical NRR is achieving stable single-atom dispersion on the substrate. As the prototype of the MXene family, Ti_3_C_2_ with O terminations have been employed in various catalysis systems serving as either catalyst or substrate.^41,42^ Moreover, Ti_3_C_2_-based SACs have been successfully fabricated for electrochemical reactions such as hydrogen evolution and water splitting.^43^ In this regard, we screen a series of transition metals, including 3d and 4d elements, supported on the Ti_3_C_2_O_2_ MXene, to investigate the feasibility of achieving single-atom dispersion. Because O terminations occupy FCC hollow sites in the Ti_3_C_2_ lattice, two possible sites for anchoring single transition metal atoms are generated (see Figure S1), namely, C site (the HCP hollow site on top of C) and M site (the FCC hollow site on top of the Ti in the first Ti atomic layer). Our calculations show that most transition metals prefer the C site, resulting in metal–oxygen bonds that stabilize single transition metal atoms (Figure S1). However, the single-atom dispersion requires not only the stable adsorption but also preventing the agglomeration.^44^ To this end, we propose to use the average adsorption energy of metal atoms in dimers to characterize the possibility of agglomeration, calculated as
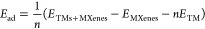
1where *E*_TMs+MXenes_, *E*_MXenes_, and *E*_TM_ represent the energy of metal atoms adsorbed on the Ti_3_C_2_O_2_, the pristine Ti_3_C_2_O_2_, and the reference energy of transition metals (see eq S1), respectively, while *n* is the number of metal atoms. Herein, the adsorption behavior of transition metal atoms is investigated under both vacuum and implicit solvent conditions. Figure 1a shows the correlation between average adsorption energy for metal dimers and adsorption energy for single metals in a vacuum. As seen, many transition metals exhibit similar adsorption energies for single atoms and dimers. For instance, the V dimer is only 0.06 eV more stable than the single V atom, indicating it is possible to obtain the coexistence of V clusters and single V atoms on the Ti_3_C_2_O_2_. Nevertheless, the possibility of agglomeration increases if there are more unpaired electrons in transition metals (gray region in Figure 1). Particularly, Ru(4d^6^) and Rh(4d^7^) are more likely to form metal clusters since the dimer adsorption is 0.54 and 0.68 eV stronger than the single-atom dispersion, respectively. On the other hand, stable single-atom adsorption can be achieved on transition metals with either more empty or fully occupied d orbitals. For example, the single-atom adsorption on the Ti_3_C_2_O_2_ for Sc(3d^1^), Zr(4d^2^), Ni(3d^8^), and Ag(4d^9^) is energetically favored. Moreover, single transition metals tend to bind stronger in the solvent. As seen in Figure 1b, most transition metals (except Ru, Rh, and Pd) exhibit stronger single-atom adsorption energies with the implicit solvent model. Consequently, the possibility to achieve single-atom dispersion is enhanced with implicit solvation. Such adsorption behavior effectively guarantees stability of the single-atom catalysts and provides unsaturated active sites for the further application in catalysis. In addition, the stability for the single-atom adsorption is assessed by the corrosion potential of TM/Ti_3_C_2_O_2_ with the implicit solvation model. The corrosion reactions of transition metals are the dissolution of transition metals to form the most stable cations/anions (eqs S9–S11) and corrosion potentials (calculated by eqs S12–S14) at pH = 0 are the highest potentials to stabilize the single-atom adsorption (Table S1). It is found that corrosion potentials for the majority of single transition metal atoms on the Ti_3_C_2_O_2_ are in the range from −1.0 to 0.2 V (vs SHE). Taking into account that the electrochemical NRR requires negative potentials, the corrosion can be effectively prevented during the NRR process.

**Figure 1:**
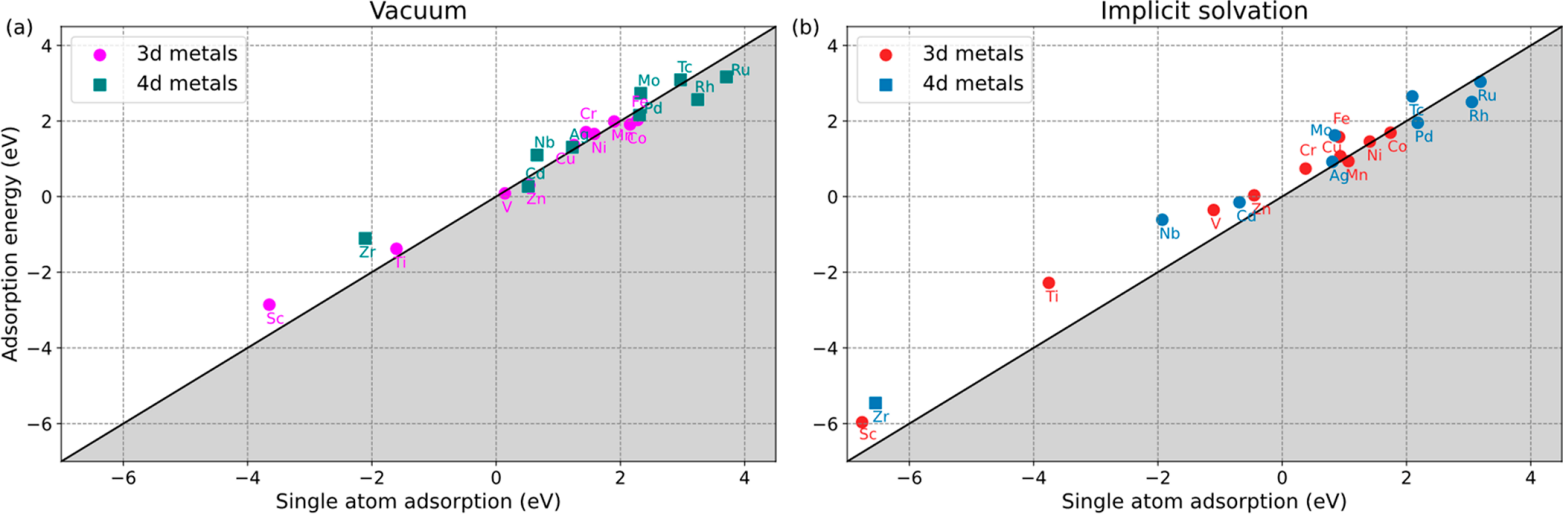
Adsorption energies of single transition metals and metal dimers on the Ti_3_C_2_O_2_ MXene in (a) a vacuum and (b) implicit solvation, calculated with eq 1.

The catalytic performance of SACs supported on the Ti_3_C_2_O_2_ can be predicted by considering the second and the third criteria given above: the adsorption behavior of the N_2_ and the possibility for NH_3_ desorption. The former reveals the feasibility for proceeding NRR, and the latter evaluates the lifecycle of the catalysts. In the electrochemical NRR, the N_2_ may adsorb on the active site in two distinct configurations: end-on and side-on (Figure S2a,b). DFT calculations show that for the majority of TMs the end-on adsorption is more energetically favored, consistent with a previous study.^45^ As seen in Figure S2c, N_2_ exhibits positive adsorption energies on transition metals with less valence electrons (Sc, Ti, Zr, Nb, and Mo), suggesting limited catalytic activity. Nevertheless, for Co, Ni, Cu, Rh, and Pd, the N_2_ can be stabilized in both end-on and side-on configurations (*E*_ad_^N_2_^ < −0.2 eV). However, further calculations indicate that the NH_3_ exhibits stronger adsorption than the most stable N_2_ adsorption configuration on all considered TM/Ti_3_C_2_O_2_ (Figure S3). Specifically, adsorption energies for NH_3_ on SACs are smaller than −1.0 eV while adsorption energies for N_2_ are in the range −0.2 to −0.8 eV (negative values refer to exothermic adsorption). Consequently, the desorption of NH_3_ would be prohibited due to the strong interactions, leading to limited activity for the transition metal atoms. Nevertheless, such an obstacle can be overcome by modifying MXene surfaces, as the catalytic performance of MXene-based catalysts is highly related to the MXene surface chemistry.^34^ Specifically, surface terminations play a vital role. In addition, it has been shown that the surface hydroxyl modification of Ti_3_C_2_ can boost electrosynthesis of ammonia, in which OH terminations can enhance the N_2_ adsorption on the Ti_3_C_2_T_2_.^46^

To this end, we have proposed a new strategy for enhancing the catalytic performance for SACs by modifying the surface chemistry of the MXene support. Instead of the fully O terminated Ti_3_C_2_ MXene, the OH terminations are introduced, except for the three most adjacent termination sites, generating the composition Ti_3_C_2_O_0.19_(OH)_1.81_, labeled as Ti_3_C_2_T_2_ (Figure 2a). The advantages for employing Ti_3_C_2_ MXene with mixed terminations are twofold. First, introducing OH termination into the Ti_3_C_2_ MXene can effectively reduce the adsorption strength of NH_3_ on the single TM atom. As shown in Figure 2b, the adsorption energies of NH_3_ have become less favorable by more than 0.5 eV with respect to that on the Ti_3_C_2_O_2_ MXene, except for Pd, Sc, and Zn (data points in the white region). Interactions between the NH_3_ and 3d metals such Ni, Co, and Fe have decreased dramatically to adsorption energies around −0.4 eV on the O/OH-terminated MXene. Notably, the NH_3_ possesses positive adsorption energies on Ag, Nb, and Mo single atoms, indicating a spontaneous desorption of NH_3_. Such weakened NH_3_ adsorption would presumably accelerate the reaction after the NH_3_ is generated. Second, the ability for single atoms to capture N_2_ molecules is enhanced by OH terminations, as shown in Figure 2c,d. The data points in the white regions are referring to transition metals that exhibit stronger N_2_ adsorption on the Ti_3_C_2_T_2_ than that of Ti_3_C_2_O_2_, indicating that the N_2_ reduction can take place easier on the TM/Ti_3_C_2_T_2_. As seen in Figure 2c,d, the adsorption N_2_ is significantly strengthened on Mo, Nb, Cr, and Ti supported on the Ti_3_C_2_T_2_, while N_2_ exhibits positive adsorption energy on the same single atoms supported on the Ti_3_C_2_O_2_. Moreover, the N_2_ exhibits stronger adsorption than that of NH_3_ on the TM/Ti_3_C_2_T_2_. In other words, a hydrogen-rich environment, which is beneficial for the reduction, has a synergetic effect on both ideal N_2_ and NH_3_ adsorption.

**Figure 2:**
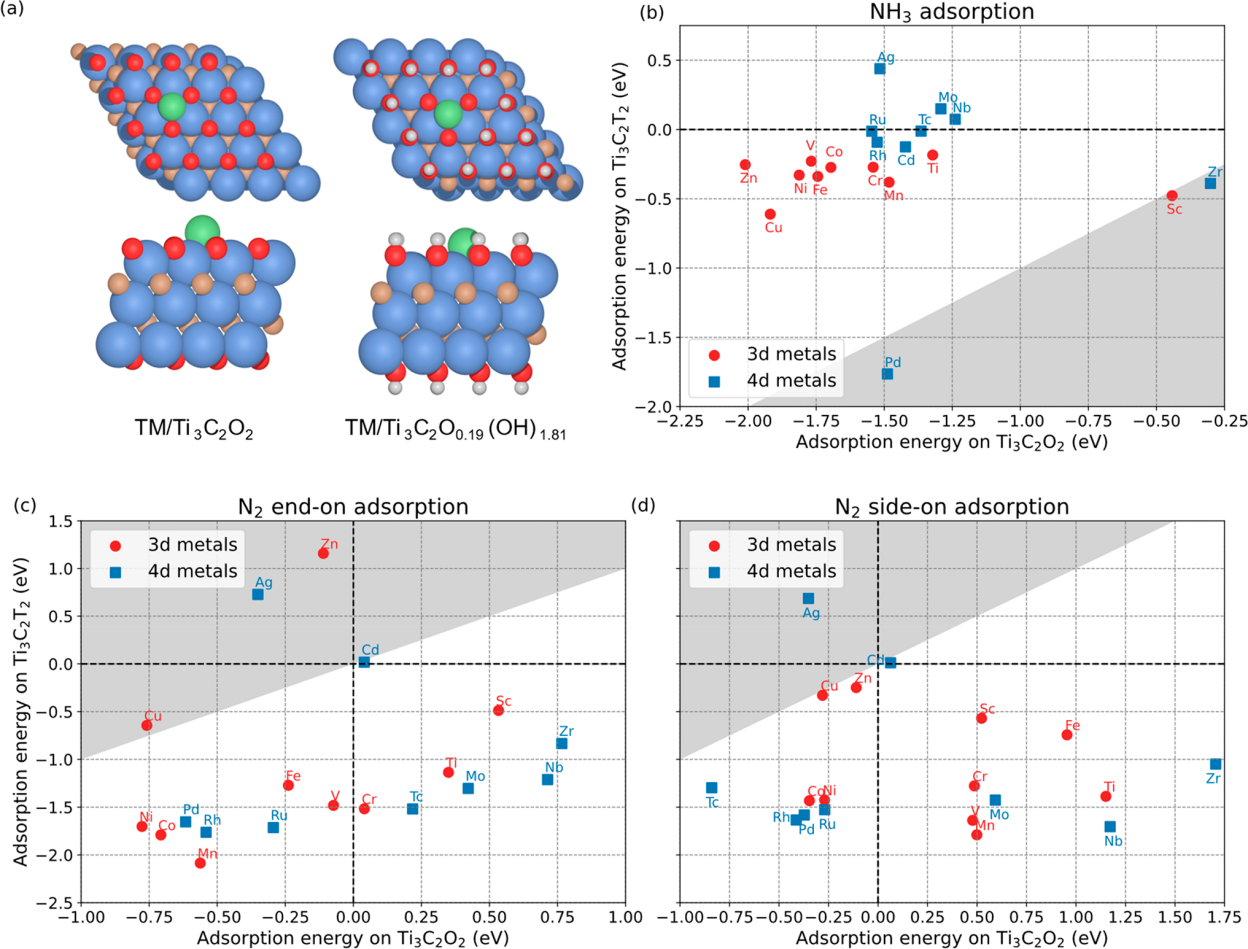
(a) Optimized structures for the TM/Ti_3_C_2_O_2_ and TM/Ti_3_C_2_O_0.19_(OH)_1.81_ (Ti_3_C_2_T_2_ for simplification). (b) Adsorption energies of the NH_3_ on the TM/Ti_3_C_2_O_2_ and TM/Ti_3_C_2_T_2_. Adsorption energies of N_2_ on the TM/Ti_3_C_2_O_2_ and TM/Ti_3_C_2_T_2_ in the (c) end-on configuration and (d) side-on configuration. The implicit solvation model is employed for all adsorption energies. The Ti, C, O, H, and TM atoms in (a) are represented by the blue, brown, red, white, and green spheres, respectively.

To this end, single atoms (Fe, Co, Ni, V, Nb, and Mo) supported on the Ti_3_C_2_T_2_ MXene can be considered as promising catalysts toward electrochemical NRR due to the desired adsorption behavior of both N_2_ and NH_3_. However, the capability to bind N_2_ for V, Mo, and Nb single atoms is highly sensitive to the amount of OH terminations on the MXene, resulting in unstable catalytic performance. Furthermore, it is noteworthy that the coadsorption of two NH_3_ molecules on the active site is considered as an important intermediate state in electrochemical NRR.^27^ Therefore, we further investigate the capability of transition metals for loading ammonia molecules by calculating the adsorption energy of the second NH_3_ on single atoms with one NH_3_ already attached according to
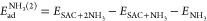
2*E*_SAC+2NH_3__, *E*_SAC+NH_3__, and *E*_NH_3__ refer to the potential energy of SACs with two NH_3_ adsorbed, SACs with only one NH_3_ attached, and a NH_3_ molecule, respectively. To achieve fast cycle for the electrochemical NRR, spontaneous desorption of the second NH_3_ is desired; that is, the adsorption energy for the second NH_3_ should be positive. However, for majority of the transition metals such as Ti, Co, Cr, Zn, and V, the second NH_3_ tends to bind even stronger than the first NH_3_ to the active site. In Figure 3, the data points in the gray region represent transition metal atoms with a stronger second NH_3_ adsorption. Such enhanced adsorption can be ascribed by the formation of hydrogen bonds that stabilize the entire system. Consequently, hydrogenation steps of N_2_ would be hindered due to occupied active sites. Despite this, the adsorption behavior of NH_3_ molecules on Fe, Pd, and Ni is desired due to weak interactions between the second NH_3_ and SACs. Of great importance, the second NH_3_ exhibits positive adsorption energy on the single Ni atom, indicating that a spontaneous desorption can take place. Taking into account that the single Ni atom exhibits stable adsorption on the Ti_3_C_2_ MXene and the desired interactions with both N_2_ and NH_3_, we therefore propose that Ni/Ti_3_C_2_T_2_ can serve as an efficient catalyst toward electrochemical NRR.

**Figure 3:**
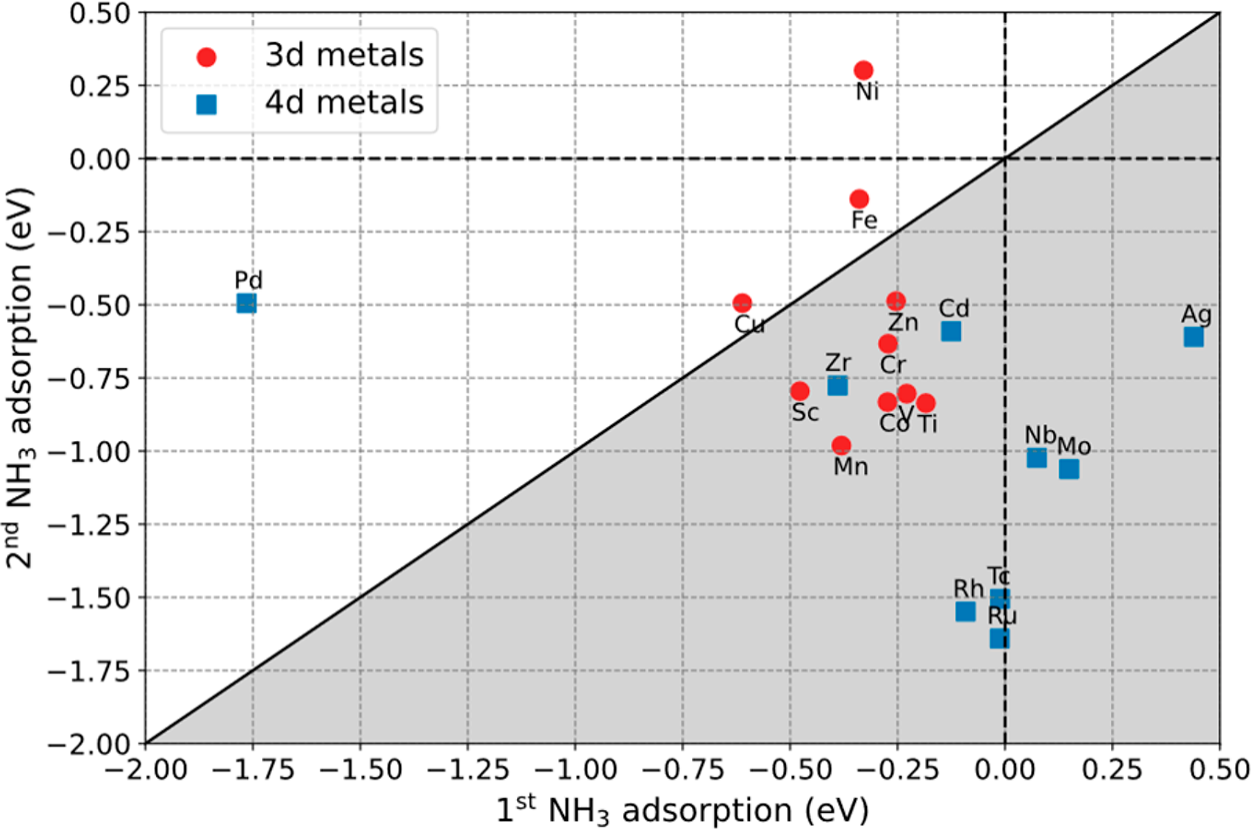
Comparison of the adsorption energy of the first NH_3_ and the second NH_3_ molecule on the TM/Ti_3_C_2_T_2_ MXene with the implicit solvation model.

As illustrated in Scheme 1, three alternative reaction pathways for electrochemical NRR have been considered on the Ni/Ti_3_C_2_T_2_ catalyst, namely, distal, alternating, and enzymatic pathway.^13^ As seen, the distal and alternating pathways (red and blue lines) initiate from the end-on adsorption of N_2_ but possess different hydrogenation sequences afterward, while the enzymatic pathway (purple lines) starts from side-on adsorption of N_2_. In the distal pathway, hydrogenations consecutively take place at the upper N until the first NH_3_ dissociates from the catalysts. In the alternating and enzymatic pathway, however, hydrogenations proceeded on both N atoms to form two NH_3_ in succession.^47^ In this work, the catalytic performance of Ni SAC is evaluated based on all three possible NRR pathways. In addition, two scenarios for hydrogenations are considered: (1) hydrogens come from the electrolyte in the form of (H^+^ + e^–^) pairs to attack N atoms, and (2) OH terminations contribute H atoms for the reduction of the nitrogen.

**Scheme 1:**
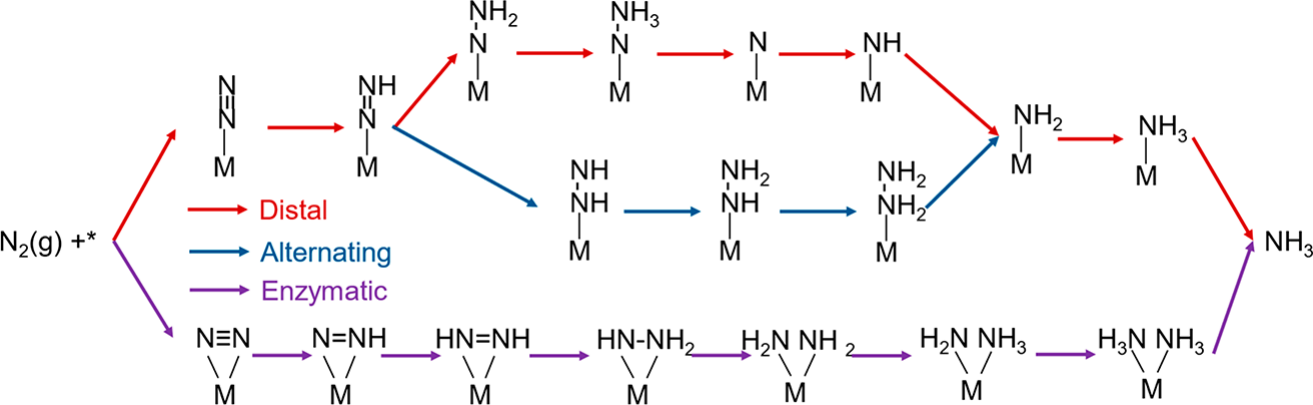
Possible Reaction Pathways for the Electrocatalytic Reduction of N_2_ to NH_3_ on Single Metal Atoms Supported on Ti_3_C_2_T_2_ MXene

We first focus on the scenario 1, in which the electrolyte serves as the source of hydrogen. Figure 4a displays the energy profiles of electrochemical NRR on the Ni/Ti_3_C_2_T_2_ catalyst. As seen, the Ni single atom supported on Ti_3_C_2_T_2_ MXene exhibits strong interactions with the N_2_ molecule. The adsorption free energies of N_2_ are −0.62 and −0.73 eV for end-on and side-on configurations with respect to SHE, which are significantly larger than other SACs.^45^ As seen, the first hydrogenation is the energy-limiting step for electrochemical NRR in the alternating and enzymatic pathways, exhibiting free energy barriers of 1.47 and 1.56 eV, respectively. Subsequent hydrogenation can proceed along the enzymatic pathway due to the low Gibbs free energy profile (overall 0.86 eV). As discussed above, the weak interaction with single NH_3_ and/or double NH_3_ on the Ni/Ti_3_C_2_T_2_, for which the relative Gibbs free energies are −0.60 and −0.28 eV, can accelerate the Ni SAC to retain the initial state for the next reduction cycle. The effect of OH terminations on the catalytic performance is elucidated by calculating the activity of the Ni/Ti_3_C_2_O_2_ catalyst. As seen in Figure 4b, the termination groups on the Ti_3_C_2_ support do not significantly influence the catalytic activity of the Ni active sites. The limiting step for the Ni/Ti_3_C_2_O_2_ catalyst remains at the first hydrogenation (N_2_ → N_2_H) with a barrier of 1.29 eV (enzymatic pathway) and 1.28 eV (distal/alternating pathway). Moreover, the second hydrogenation step along the enzymatic pathway exhibits an exothermic characteristic on the Ni/Ti_3_C_2_T_2_, while a Gibbs free energy barrier of 0.14 eV should be overcome on the Ni/Ti_3_C_2_O_2_ (Figure 4b). Such a discrepancy in Gibbs free energy profiles indicates that the single Ni atom exhibits different selectivity on the Ti_3_C_2_T_2_ and Ti_3_C_2_O_2_. As a result, the enzymatic pathway is no longer energetically favored while the alternating pathway exhibits the lowest overall Gibbs free energy of 1.05 eV on the Ni/Ti_3_C_2_O_2_. However, the catalytic performance of the Ni/Ti_3_C_2_O_2_ catalyst is profoundly limited by the strong interactions between the single Ni atom and NH_3_ molecules (*G*(NH_3_) = −1.60 eV and *G*(N_2_H_6_) = −1.51 eV). Such a strong adsorption of synthesized NH_3_ would hinder further reactions. Furthermore, our calculations show that the Ni/Ti_3_C_2_T_2_ exhibits better catalytic activity under implicit solvation condition. As seen in Figure S4, the limiting step for the Ni/Ti_3_C_2_T_2_ along the enzymatic pathway is decreased from 1.56 to 1.36 eV. In addition, the relative Gibbs free energy of *NHNH is reduced to −0.11 eV in solution, indicating a more stable intermediate. Of importance, the accelerated reaction kinetics can be expected in the solution because the relative Gibbs free energy of N_2_H_6_ has been increased from −0.28 eV (in vacuum) to −0.04 eV (in implicit solvation).

**Figure 4:**
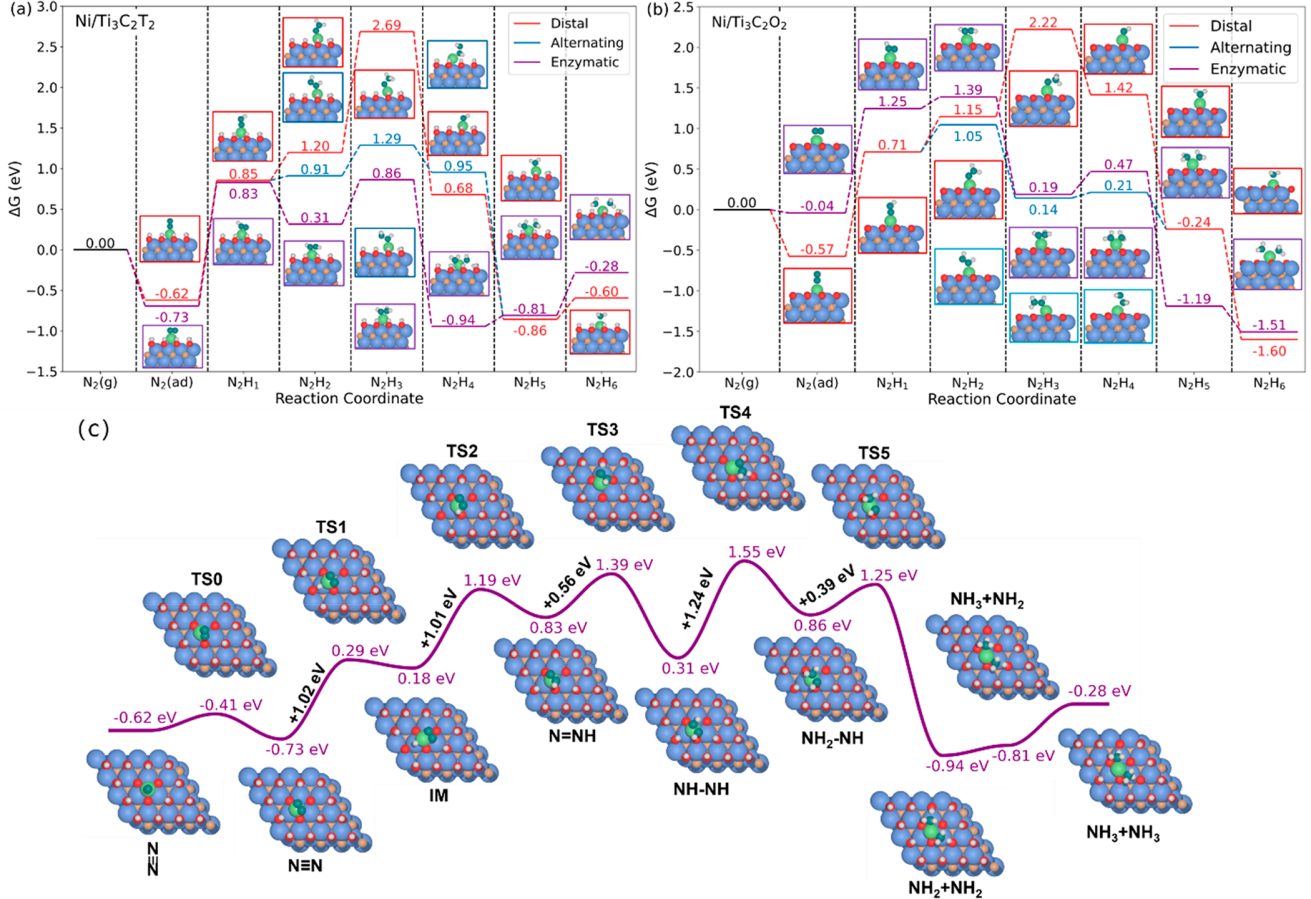
Reaction pathways and Gibbs free energy profiles for the nitrogen fixation on the (a) Ni/Ti_3_C_2_T_2_ and (b) Ni/Ti_3_C_2_O_2_ catalysts. (c) Enzymatic pathway and corresponding Gibbs free energy profiles for NRR on Ni/Ti_3_C_2_T_2_. The Ti, O, C, H, N, and Ni atoms are represented by the blue, red, brown, white, light green, and dark green circles, respectively.

Alternatively, the OH terminations can provide the H atoms for NRR (scenario 2). Full Gibbs free energies and geometry structure of all species including transition states for NRR via the enzymatic pathway are presented in Figure 4c. In this work, hydroxyl groups are assumed to be re-formed after each hydrogenation step. However, the first hydrogenation step requires high-energy input (2.03 eV for passing through TS1 and TS2). Such a high-energy requirement can be ascribed to the stability of OH terminations on the Ti_3_C_2_ MXene.^48^ Nevertheless, such alternative reaction mechanism provides potential pathways for achieving NH_3_ fixation via heterogeneous catalysis.

As a final remark, F terminations are also commonly observed in experiments. However, additional calculations show that F terminations exhibit limited promotion on the catalytic performance of single Ni atoms (Figure S5 and Table S2). Similar results can be observed for other terminations including Cl, Br, I, and S. Interestingly, the Ni/Ti_3_C_2_O_0.19_Te_1.81_ exhibits positive adsorption energies for the second NH_3_, suggesting promising reaction kinetics for electrochemical NRR. However, the synthesis of multilayers of the Te-terminated Ti_3_C_2_ requires high temperatures (300–600 °C),^49^ and the fabrication of the Ti_3_C_2_Te_2_ monolayer has not been reported yet. Such obstacles would therefore hinder the further application of Te-terminated Ti_3_C_2_ in single-atom catalysis. Consequently, it is important to maintain a considerable concentration of OH terminations to obtain high efficiency.

In conclusion, on the basis of first-principles calculations, we investigated the potential of a single transition metal atom anchored on the Ti_3_C_2_T_2_ (T = O and/or OH) MXene as electrocatalyst for NRR and the effect OH terminations on the catalytic performance. Our computational screening of anchoring single TM atoms on the Ti_3_C_2_T_2_ MXene has shown that a single atom supported on Ti_3_C_2_O_2_ MXene can bind N_2_ with considerable adsorption energy but suffer from the strong interaction with NH_3_, leading to limited life cycle. Importantly, we reveal that introducing OH terminations to Ti_3_C_2_ MXene can effectively enhance the N_2_ adsorption and suppress the NH_3_ adsorption. Furthermore, an in-depth understanding for the NH_3_ adsorption was obtained by considering the coadsorption of two NH_3_ molecules. Our DFT calculations predict that the single Ni atom supported on Ni/Ti_3_C_2_O_0.19_(OH)_1.81_ is a promising SAC toward electrochemical NRR among all considered systems. Subsequent calculations indicate the existence of OH terminations will not significantly influence the catalytic performance of the single Ni atom but accelerate reduction reactions by weakening the binding energy of NH_3_. We anticipate that our study can provide fast screening criteria for evaluation the catalytic performance of MXene-based SACs and a comprehensive understanding on effects of surface chemistry on their performance in the electrochemical processes of NRR.

## Supporting Information Available

Computational parameters and methods; adsorption of single transition metal atoms on the Ti_3_C_2_O_2_; corrosion potentials for transition metal atoms on the Ti_3_C_2_O_2_; adsorption behavior of the N_2_ molecule on the TM/Ti_3_C_2_O_2_ with the implicit solvation model; adsorption of the NH_3_ on the TM/Ti_3_C_2_O_2_ with the implicit solvation model; implicit solvation effect on the electrochemical NRR on the Ni/Ti_3_C_2_T_2_ along the enzymatic pathway; influence of other terminations including F, Cl, Br, I, S, and Te on the catalytic performance of the Ni SAC (PDF)
Transparent Peer Review report available (PDF)
