# Supplementary material for: Termination-Accelerated Electrochemical Nitrogen Fixation on Single-Atom Catalysts Supported by MXenes
Source: J Phys Chem Lett. 2022 Mar 23;13(12):2800–7. doi: 10.1021/acs.jpclett.2c00195 (PMC8978179; doi:10.1021/acs.jpclett.2c00195)
Supplement: Supplementary file 1 — jz2c00195_si_001.pdf [file jz2c00195_si_001.pdf]

Supporting Information

Termination-Accelerated Electrochemical Nitrogen Fixation on Single-Atom Catalysts Supported by MXenes

Kaifeng Niu[1,2], Lifeng Chi[2*], Johanna Rosen[1], Jonas Björk[1*]

[1]Department of Physics, Chemistry and Biology, IFM, Linköping University, 581 83 Linköping, Sweden

[2]Institute of Functional Nano & Soft Materials (FUNSOM) and Jiangsu Key Laboratory for Carbon-Based Functional Materials & Devices, Soochow University, Suzhou 215123, P. R. China

Lifeng Chi: chilf@suda.edu.cn

Jonas Björk: jonas.bjork@liu.se

1. Computational methods

All spin-polarized density functional theory calculations were performed by Vienna ab-initio simulation package (VASP) and atomic simulation environment.[1,2] The projector augmented wave potentials were employed to describe the electron-ion interactions and the van der Waals density functional (vdWDF) in a version of rev-vdWDF2 proposed by Hamad was used to treat exchange-correlationi interactions.[3,4] The cutoff energy for the plane wave basis was set as 400 eV. A 20 Å vacuum layer was adopted to avoid periodic image interactions. The $p(4 \times 4)$ Ti_3C_2 MXene supercells with O and OH terminal groups were employed as the support of the single transition metal atoms. A 4 × 4 × 1 grid was used to model the Brillouin zone. A force tolerance of 0.02 eV/Å was adopted for all calculations.

The stability and the possibility for agglomeration of transition metal atoms were characterized by the adsorption energy with respect to the energy of cooresponding metal atoms from their bulk counterparts. The energy for transition metal atoms was calculated by:

$$E_{TM} = \frac{1}{m} \times E_{TM\,primitive\ cell}, \tag{S1}$$

in which the $E_{TM\,primitive\ cell}$ is the energy for the primitive cell of the metal lattice, and m is the number of atoms in the primitive cell.

The computational hydrogen electrode (CHE) method proposed by Nørskov et al is employed to calculate Gibbs free energy profiles for electrochemical NRR,[5] in which the electrochemical nitrogen reduction reaction is: $N_2(g) + 6H^+ + 6e^- = 2NH_3$, including 6 proton- electron pair ($H^+ + e^-$) transfer steps. The change of Gibbs free energy (ΔG) for each elementary step at zero potential was calculated as

$$\Delta G = \Delta E + \Delta H^{vib} - T\Delta S^{vib} + \Delta G_{pH}, \tag{S2}$$

where E is the potential energy. H^{vib} is the vibrational enthalpy, which is given as

$$H^{vib}(T) = k_B \sum_i \left(\frac{h\nu_i}{2k_B} + \frac{h\nu_i}{k_B T}\frac{1}{e^{\frac{h\nu_i}{k_B T}}-1}\right), \tag{S3}$$

and S^{vib} is the vibrational entropy

$$S^{vib}(T) = k_B \sum_i \left(\frac{h\nu_i}{k_B T}\frac{1}{e^{\frac{h\nu_i}{k_B T}}-1} - \ln\left(1 - e^{-\frac{h\nu_i}{k_B T}}\right)\right), \tag{S4}$$

where k_B is Boltzmann's constant and h is Planck's constant. ν_i refers to the vibrational frequency of eigenmode i. Vibrational eigenmodes were obtained within the harmonic approximation.[6] According to the CHE model, the chemical potential of the H^+/e^- pair under standard conditions is equal to the half of the Gibbs free energy of the H_2 (pH = 0, p = 1 bar, T = 298.15 K). The Gibbs free energy of H_2 is defined by:

$$G_{H_2}(p,T) = H_{H_2}^{elec} + H_{H_2}^{trans}(T) + H_{H_2}^{rot}(T) + H_{H_2}^{vib}(T) - TS_{H_2}(p,T), \tag{S5}$$

where $H_{H_2}^{elec}$, $H_{H_2}^{trans}(T)$, $H_{H_2}^{rot}(T)$, and $H_{H_2}^{vib}(T)$ are the electronic, translational, vibrational, and rotational enthalpies of H_2, respectively. The translational enthalpy is defined by:

$$H_{H_2}^{trans}(T) = \frac{3}{2}k_B T, \tag{S6}$$

the rotational enthalpy for H_2 is defined by:

$$H_{H_2}^{rot}(T) = \frac{3}{2}k_B T, \quad \text{(S7)}$$

the vibrational enthalpy and entropy are defined the same as eq. S3. The tabulated value of entropy of the H_2 was used.[7]

The ΔG_{pH} is the free energy contributions related to the H^+ concentration,[8] which is given as

$$\Delta G_{pH} = \ln(10) \times k_B \times pH \quad \text{(S8)}$$

In this work, the pH was set as 0 and the temperature was set as 298.15 K to represent the acidic media and room temperature.

The transition states search for elementary hydrogenation steps was first calculated by the Climb-image Nudged Elastic Band method (CI-NEB), where 10 images were inserted between two N_xH_y (x = 1 or 2; 0 ≤ y ≤ 6).[9] The central image were subsequently employed as the input of Dimer method to obtain accurate transition states.[10] Only one imageinary frequency was obtained at each saddle point. The solvation effect on adsorption energies for transition metal atoms, N_2, and NH_3 were calculated by VASPsol.[11]

2. Single atom adsorption on the $Ti_3C_2O_2$

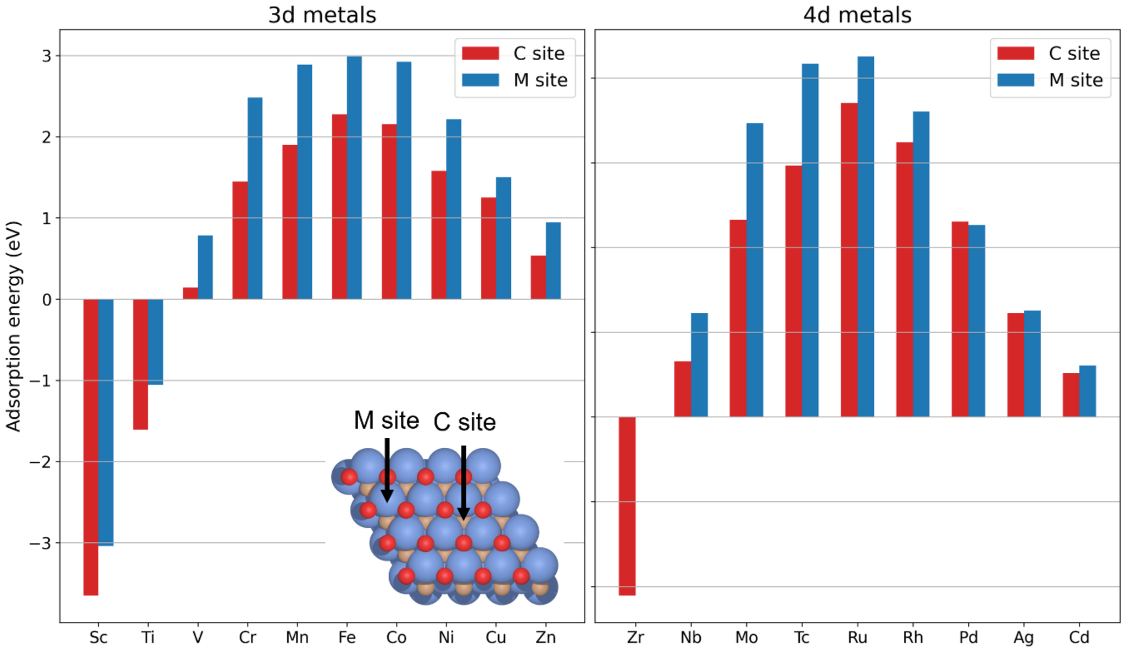

Figure S1. The adsorption energies for single metal atoms on different sites of the $Ti_3C_2O_2$, calculated by eq. (1) in the manuscript. Two possible sites are given in the inserted figure. The C, O, and Ti atoms are represented by the brown, red, and blue circles, respectively.

3. The stability of the single-atom adsorption on $Ti_3C_2O_2$.

Table S1. The adsorption energies and corrosion potentials for stabilizing the single atoms.

Metal	Adsorption energy E_{ad} (eV)	Corrosion potential U (vs. SHE at pH=0)	Reference ion
Sc	-6.760	0.227	Sc^{3+}
Ti	-3.755	0.043	Ti^{3+}
V	-1.103	-0.576	V^{2+}
Cr	0.377	-1.043	Cr^{2+}
Mn	1.066	-1.715	Mn^{2+}
Fe	0.916	-0.867	Fe^{2+}
Co	1.743	-1.154	Co^{2+}
Ni	1.409	-0.941	Ni^{2+}
Cu	0.936	-0.418	Cu^{+}
Zn	-0.451	-0.537	Zn^{2+}
Zr	-6.545	0.191	Zr^{4+}
Nb	-1.930	-0.193	$HNbO_3$
Mo	0.848	0.006	$HMoO_4^-$
Tc	2.093	/	/
Ru	3.190	-0.816	Ru^{2+}
Rh	3.053	-0.926	Rh^{2+}
Pd	2.181	-0.176	Pd^{2+}
Ag	0.808	-0.009	Ag^{+}
Cd	-0.688	-0.058	Cd^{2+}

The corrosion potential of single transition metals on the $Ti_3C_2O_2$ in the electrochemical environment (pH = 0) has been evaluated according to dissolution reactions, which are defined as:
for metal cations:

$$M(ad) \rightarrow M^{n+} + ne^-, \tag{S9}$$

for Nb, the $HNbO_3$ is the most stable form:

$$Nb(ad) + 3H_2O \rightarrow HNbO_3 + 5H^+(aq) + 5e^-, \tag{S10}$$

and for Mo, the $HMoO_4^-$ anion is the most stable:

$$Mo(ad) + 4H_2O \rightarrow HMoO_4^- + 7H^+(aq) + 6e^-. \tag{S11}$$

Correspondingly, the corrosion potential for transition metals is defined as:

$$U(M) = -\frac{E_{ad}(M) - E_{formation}(m^{n+})}{ne}, \tag{S12}$$

$$U(Nb) = -\frac{E_{ad}(Nb) - E_{formation}(HNbO_3) + 3E(H_2O)}{5e}, \tag{S13}$$

and

$$U(Mo) = -\frac{E_{ad}(Mo) - E_{formation}(HMoO_4^-) + 4E(H_2O)}{7e}. \tag{S14}$$

in which $E_{ad}(M)$, $E_{formation}(m^{n+})$ and *n* refer to the adsorption energy of single M atom on the $Ti_3C_2O_2$ in solution (the free energy for solid phases are estimated by the electronic enthalpy at 0 K), the formation energy of metal cations[12,13] obtained from the dissolution potential of pure metals in experiment as tabulated in Ref. [12] and Ref. [13], and the number of positive charges of the cation, respectively. Note that the formation free energy of the H^+ ions are not included in the corrosion potentials since it is zero by definition for pH = 0.

4. The adsorption on N_2 on TM/$Ti_3C_2O_2$.

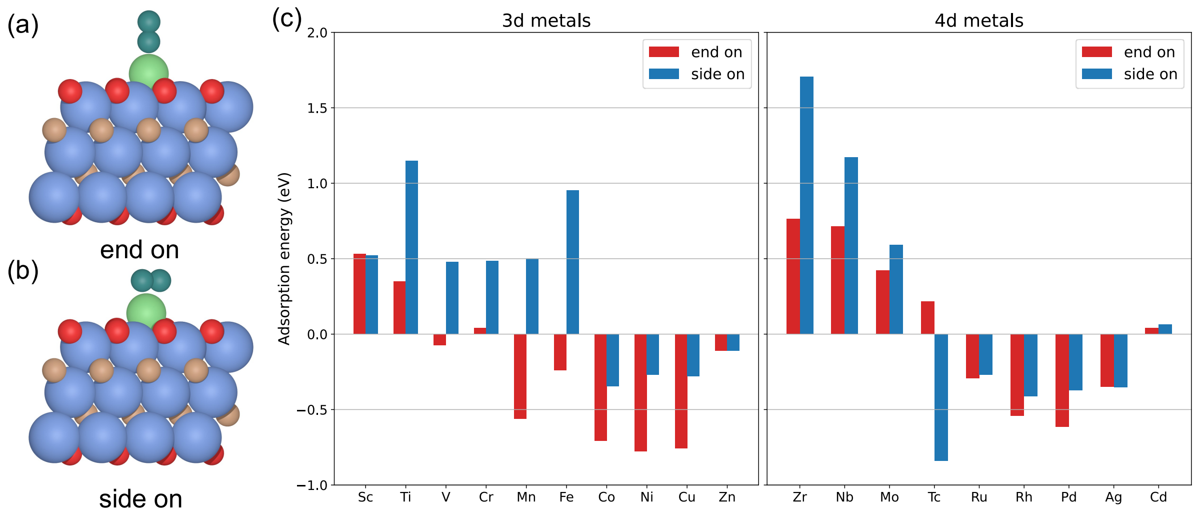

Figure S2. Optimized structures for N_2 adsorption on the SAC in (a) end-on configuration and (b) side-on configuration. (c) The adsorption energies of the N_2 on SACs with implicit solvation model. Ti, C, O, N, and TMs in (a) and (b) are represented by the blue, brown, red, dark green, and light green circles, respectively.

The adsorption energy of N_2 on the SACs is calculated by:

$$E_{ad}^{N_2} = E_{SAC+N_2} - E_{SAC} \; - \; E_{N_2} \quad (S15)$$

in which E_{SAC+N_2}, E_{SAC}, and E_{N_2} represent the potential energy of SACs with N_2 adsorbed, the pristine SACs, and the N_2 molecule, respectively.

5. The adsorption of NH_3 on the TM/$Ti_3C_2O_2$.

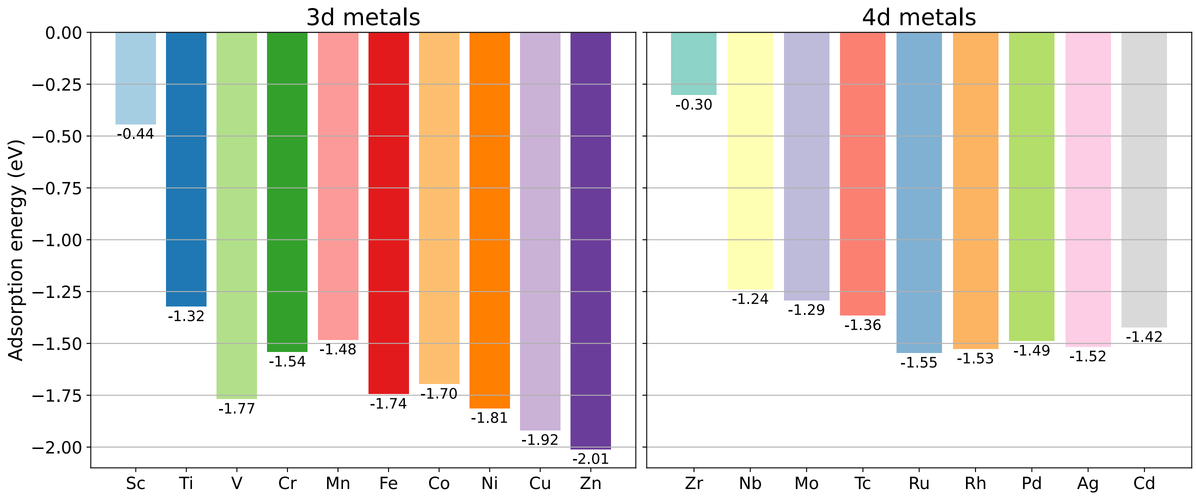

Figure S3. The adsorption energy of NH_3 on the TM/$Ti_3C_2O_2$ MXenes with implicit solvation model.

The adsorption energy of NH_3 on SACs is calculated by:

$$E_{ad}^{NH_3} = E_{SAC+NH_3} - E_{SAC} - E_{NH_3} \quad \text{(S16)}$$

in which E_{SAC+NH_3}, E_{SAC}, and E_{NH_3} represent the potential energy of SACs with NH_3 adsorbed, the pristine SACs, and the NH_3 molecule, respectively.

6. The Gibbs free energy profile for enzymatic pathway.

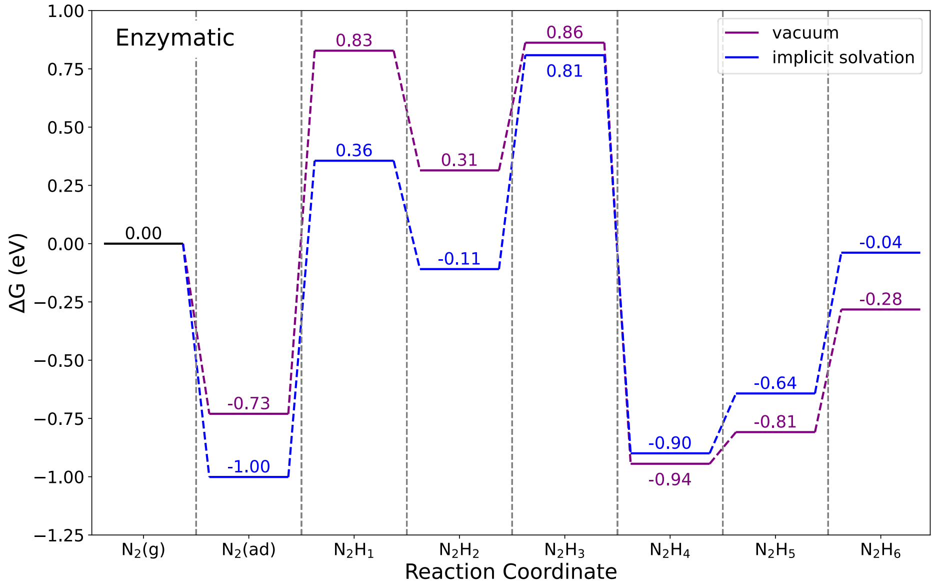

Figure S4. The Gibbs free energy profile for the electrochemical NRR on Ni/$Ti_3C_2T_2$ along the enzymatic pathway in vacuum (purple) and with implicit solvation (blue).

Figure S4 shows the influence of the implicit solvation effect on the catalytic performance of Ni active sites. As seen, the N_2 adsorption is more stable in the solution than in vacuum. The limiting Gibbs free energy barrier has been decreased from 1.56 eV to 1.36 eV, indicating the catalytic performance of the Ni atom is promoted in the solvent condition. In addition, reaction intermediate states such as *NNH and *NHNH bind stronger to the Ni in the solution, leading to a promotion of reaction kinetics. Furthermore, the implicit solvation effect exhibits positive effect on the adsorption of NH_3, in which the Gibbs free energy of co-adsorption of two NH_3 molecules (N_2H_6) is decreased from -0.28 eV to -0.04 eV. Such weak adsorption of NH_3 indicates the Ni active sites can be available rapidly, resulting in accelerated reaction kinetics.

7. The influence of other possible terminations

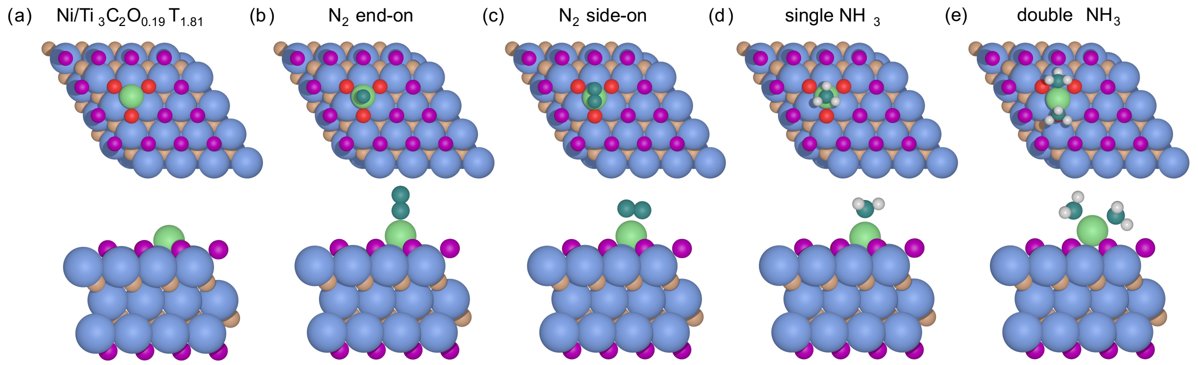

Figure S5. The optimized structures (top view for the upper panel and side view for the lower panel) for (a) $Ni/Ti_3C_2O_{0.19}T_{1.81}$ (T = F, Cl, Br, I, S and Te), (b) the end-on adsorption of N_2, (c) the side-on adsorption of N_2, (d) single NH_3 adsorption, and (e) double NH_3 adsorption on the $Ni/Ti_3C_2O_{0.19}T_{1.81}$. The Ni, O, Ti, C, T, and N atoms are represented by the light green, red, blue, brown, purple, and dark green circles, respectively.

Table S2. The adsorption of Ni, N_2, and NH_3 on $Ni/Ti_3C_2T_2$ MXenes with different terminations in vacuum.

	Ni adsorption (eV)	N_2 adsorption (eV)		NH_3 adsorption (eV)	
		end on	side on	Single NH_3	2nd NH_3
$Ni/Ti_3C_2O_2$	1.57	-0.93	-0.44	-1.42	/
$Ni/Ti_3C_2O_{0.19}(OH)_{1.81}$	2.03	-1.39	-1.16	-0.45	0.15
$Ni/Ti_3C_2O_{0.19}F_{1.81}$	1.95	-0.99	-0.51	-1.21	-0.46
$Ni/Ti_3C_2O_{0.19}Cl_{1.81}$	1.92	-1.01	-0.64	-1.15	-1.08
$Ni/Ti_3C_2O_{0.19}Br_{1.81}$	1.95	-1.03	-0.55	-1.06	-1.00
$Ni/Ti_3C_2O_{0.19}I_{1.81}$	1.93	-1.02	-0.75	-0.96	/[a]
$Ni/Ti_3C_2O_{0.19}S_{1.81}$	1.52	-0.95	-0.74	-1.48	-1.96
$Ni/Ti_3C_2O_{0.19}Te_{1.81}$	1.84	-0.99	-0.86	-1.03	0.21

[a]No stable adsorption configuration

In order to understand the effects of other termination groups on the catalytic performance, the catalytic performance of single Ni atom supported on $Ti_3C_2O_{0.19}T_{1.81}$ (T = F, Cl, Br, I, S, and Te) MXenes are investigated based on proposed screening criteria. As listed in Table S2, $Ni/Ti_3C_2O_{0.19}S_{1.81}$ exhibits similar catalytic performance as the $Ni/Ti_3C_2O_2$, in which strong NH_3 adsorption would prohibit further catalytic cycles. In addition, halogen terminated Ti_3C_2 MXenes exhibit weaker Ni adsorption but can enhance the N_2 adsorption comparing to the O terminated Ti_3C_2. Nevertheless, the potential catalytic performance for $Ni/Ti_3C_2O_{0.19}X_{1.81}$ (X = F, Cl, Br, and I) is limited by the strong NH_3 adsorption. Interestingly, the $Ni/Ti_3C_2O_{0.19}Te_{1.81}$ exhibits positive adsorption energies for the 2nd NH_3 (0.21 eV), suggesting promising reaction kinetics for electrochemical NRR. However, practical challenges for multilayers of Te terminated Ti_3C_2 MXenes ($Ti_3C_2Te_2$) remain at the harsh synthetic conditions (300°C to 600°C in molten alkali metal halides).[14] Furthermore, the fabrication of the $Ti_3C_2Te_2$ monolayer has not been reported yet, which prevents further applications in single atom catalysis.

References

(1) Kresse, G.; Furthmuller, J. Efficient Iterative Schemes for Ab Initio Total-Energy Calculations Using a Plane-Wave Basis Set. *Phys. Rev. B* **1996**, *54* (16), 11169–11186.

(2) Hjorth Larsen, A.; Jorgen Mortensen, J.; Blomqvist, J.; Castelli, I. E.; Christensen, R.; Dulak, M.; Friis, J.; Groves, M. N.; Hammer, B.; Hargus, C.; Hermes, E. D.; Jennings, P. C.; Bjerre Jensen, P.; Kermode, J.; Kitchin, J. R.; Leonhard Kolsbjerg, E.; Kubal, J.; Kaasbjerg, K.; Lysgaard, S.; Bergmann Maronsson, J.; Maxson, T.; Olsen, T.; Pastewka, L.; Peterson, A.; Rostgaard, C.; Schiotz, J.; Schutt, O.; Strange, M.; Thygesen, K. S.; Vegge, T.; Vilhelmsen, L.; Walter, M.; Zeng, Z.; Jacobsen, K. W. The Atomic Simulation Environment-a Python Library for Working with Atoms. *J. Phys. Condensed Matter.* **2017**, *29* (27), 273002.

(3) Blöch. Projector Agumented-Wave Method. *Phys. Rev. B* **1994**, *50*(24).

(4) Hamada, I. Van Der Waals Density Functional Made Accurate. *Phys. Rev. B* **2014**, *89* (12), 121103.

(5) Norskov, J. K.; Rossmeisl, J.; Logadottir, A.; Lindqvist, L.; Kitchin, J. R.; Bligaard, T.; Jonsson, H. Origin of the Overpotential for Oxygen Reduction at a Fuel-Cell Cathode. *J. Phys. Chem. B* **2004**, *108* (46), 17886–17892.

(6) Björk, J. Thermodynamics of an Electrocyclic Ring-Closure Reaction on Au(111). *J. Phys. Chem. C* **2016**, *120* (38), 21716–21721.

(7) Chase, M. W. NIST-JANAF Thermochemical Tables. *J. Phys. Chem. Ref. Data* **1998**, *9*, 1310.

(8) Liu, F.; Song, L.; Liu, Y.; Zheng, F.; Wang, L.; Palotás, K.; Lin, H.; Li, Y. Using the N-N Dipole as a Theoretical Indicator for Estimating the Electrocatalytic Performance of Active Sites in the Nitrogen Reduction Reaction: Single Transition Metal Atoms Embedded in Two Dimensional Phthalocyanine. *J Mater. Chem A* **2020**, *8* (7), 3598–3605.

(9) Henkelman, G.; Uberuaga, B. P.; Jonsson, H. A Climbing Image Nudged Elastic Band Method for Finding Saddle Points and Minimum Energy Paths. *J. Chem. Phys.* **2000**, *113* (22), 9901–9904.

(10) Henkelman, G.; Jonsson, H. A Dimer Method for Finding Saddle Points on High Dimensional Potential Surfaces Using Only First Derivatives. *J. Chem. Phys.* **1999**, *111* (15), 7010–7022.

(11) Mathew, K.; Sundararaman, R.; Letchworth-Weaver, K.; Arias, T. A.; Hennig, R. G. Implicit Solvation Model for Density-Functional Study of Nanocrystal Surfaces and Reaction Pathways. *J. Chem. Phys.* **2014**, *140* (8), 084106.

(12) Wagman, D. D.; Evans, W. H.; Parker, V. B.; Schumm, R. H.; Halow, I.; Bailey, S. M.; Churney, L.; Nuttall, R. L. The NBS Tables of Chemical Thermodynamic Properties: Selected Values for Inorganic and C_1 and C_2 Organic Substances in SI Unit. *J. Phys. Chem. Ref. Data* **1982**.

(13) Pourbaix, M. *Atlas of Electrochemical Equilibria in Aqueous Solutions*; National Association of Corrosion Engineers: Houston, Texas, 1974.

(14) Kamysbayev, V.; Filatov, A. S.; Hu, H.; Rui, X.; Lagunas, F.; Wang, D.; Klie, R. F.; Talapin, D. V. Covalent Surface Modifications and Superconductivity of Two-Dimensional Metal Carbide MXenes. *Science* **2020**, *369* (6506), 979–983.
