# Supplementary material for: Termination-Accelerated Electrochemical Nitrogen Fixation on Single-Atom Catalysts Supported by MXenes
Source: J Phys Chem Lett. 2022 Mar 23;13(12):2800–7. doi: 10.1021/acs.jpclett.2c00195 (PMC8978179; doi:10.1021/acs.jpclett.2c00195)
Supplement: Supplementary file 2 — jz2c00195_si_002.pdf [file jz2c00195_si_002.pdf]

jz-2022-00195e.R1

Name: Peer Review Information for "Termination-Accelerated Electrochemical Nitrogen Fixation on Single-Atom Catalysts Supported by MXenes"

First Round of Reviewer Comments

Reviewer: 1

Comments to the Author

This work reported the transition metal atoms supported on MXene with O/OH terminations, as single atom catalyst (SAC) for electrochemical nitrogen reduction. They found that the OH terminations on $Ti_3C_2T_2$ MXene can effectively enhance the N_2 adsorption and decrease the NH_3 adsorption for single atoms. This study would provide fast screening criteria for evaluation the catalytic performance of MXene-based SACs and a comprehensive understanding on effects of surface chemistry on their performance in the electrochemical processes of NRR. It is interesting and I suggest its acceptance after minor revision.

1. As I found that a similar work was published recently (Journal of Colloid and Interface Science, 2022, 605, 897–905 898). I suggest that the authors should compare their study with this published work and discussion more about their novelty. In addition, the performance should be compared with other materials to highlight the novelty of this work, such as 10.1021/acsnano.1c08109 ; 10.1002/aenm.202101699 .

2. When calculation the change of Gibbs free energy for each elementary step at zero potential, the changes of the zero-point energy are usually applied. However, in supporting information, I didn't find this part in calculation section.

3. How about the effect of other termination group such as Cl, Br, I, S and Te on catalytic activity for SAC-supported MXene?

4. From Figure 4, it is clear that the O/OH terminations can not only change the energy of the N2 hydrogenation at the first step, it also has an importance effect on other steps via different reaction pathway. Therefore, more discussion should be added to focus on each reaction step.

Reviewer: 2

Comments to the Author

Niu et al. present a systematic computational search for single-atom catalysts for nitrogen reduction supported on MXene substrates. Specifically, they compare adsorption energies of single metal atoms vs dimers as a proxy for obtaining well-dispersed catalyst atoms on the substrates, and those of nitrogen vs ammonia to identify candidates that strongly bind the reactant, but can release the product. Based on these metrics, they identify Ni supported on the Ti3C2T2 MXene as the best candidate, and map out the energetics of the nitrogen reduction reaction on this material in detail. Overall, the study is well-organized, systematically scanning the complex space of materials to find a promising catalyst, and is well-written, meriting publication in JPCL.

It would strengthen the manuscript to further justify the choice of adsorption energies as a metric for the catalyst stability. In particular, all computed energies are for neutral adsorbates in vacuum. This could change substantially in the electrochemical environment, especially given the potentially harsh potentials required for nitrogen reduction. Similarly, the reaction analysis is based on proton-coupled electron transfer steps, which could be a poor approximation for several of the nitrogen reduction intermediates. Qualifying the results shown in the manuscript to indicate these potentially important effects would be useful.

Reviewer: 3

Comments to the Author

The authors investigated the potential of a single transition metal atom anchored on the Ti3C2T2 (T = O and/or OH) MXene as electrocatalyst for NRR and the effect OH terminations on the catalytic performance. While a key point is that the adsorption energy of single transition metal Ni on the Ti3C2O2 MXene is positive, see fig.1 and formula (1), that means Ni is not adsorbed on the surface of Ti3C2O2, based on this positive adsorption energy, we do not have any next step for NRR or other electrocatalysis performances on Ni@ti3C2O2 system. This paper is not recommended because it does not provide correct physical insights.

Author's Response to Peer Review Comments:

Dear Prof. Editor,

thank you for communicating the reviewers' overall positive comments of our work. On the following pages, we give point-to-point replies addressing all the comments and questions and detail the changes we have made to the manuscript. In addition, we have attached versions of the main manuscript and supporting information with changes indicated in red font.

We hope that you find the revision satisfactory and our work suitable for publication in *JPCL*.

Sincerely yours,

Jonas Björk, on behalf of the co-authors

Reviewer 1:

This work reported the transition metal atoms supported on MXene with O/OH terminations, as single atom catalyst (SAC) for electrochemical nitrogen reduction. They found that the OH terminations on $Ti_3C_2T_2$ MXene can effectively enhance the N_2 adsorption and decrease the NH_3 adsorption for single atoms. This study would provide fast screening criteria for evaluation the catalytic performance of MXene-based SACs and a comprehensive understanding on effects of surface chemistry on their performance in the electrochemical processes of NRR. It is interesting and I suggest its acceptance after minor revision.

Response: We thank the reviewer for the many suggestions which helped us improve our manuscript.

1. As I found that a similar work was published recently (Journal of Colloid and Interface Science, 2022, 605, 897–905 898). I suggest that the authors should compare their study with this published work and discussion more about their novelty. In addition, the performance should be compared with other materials to highlight the novelty of this work, such as 10.1021/acsnano.1c08109; 10.1002/aenm.202101699.

Response: In the suggested study [*J. Colloid Interface Sci.* **2021**, *605*, 897], Ge et al have investigated the catalytic performance of single transition metal atom decorated *v*-Mo_2CT_z MXenes, in which the single transition metal atoms are anchored at the O vacancy on the Mo_2CT_z MXenes. Furthermore, the electrochemical nitrogen reduction is considered on the Ir@*v*-Mo_2CO_2. The importance of the suggested paper has been emphasized in the manuscript (citation [34]). In our study, the screening of promising transition metals is based on the stability of anchoring transition metal atoms in the form of single-atom adsorption, the capability for metal atoms to capture N_2, and the ability for transition metals to release the NH_3. Taking into consideration that the electrochemical NRR can be proceeded along multiple pathways, we have investigated the adsorption behavior of two NH_3 molecules in order to obtain a complete understanding for the catalytic performance of transition metals. Furthermore, we not only study the single transition metal atoms on the $Ti_3C_2O_2$ MXenes, but also focus on the influence of the OH terminations on the catalytic performance. In this work, we have shown that OH terminations will not necessarily reduce the catalytic activity of the single atom. On the contrary, the existence of the OH terminations can effectively reduce the adsorption of NH_3 on the single atoms, which would significantly accelerate the reaction kinetics. Based on the proposed criteria, we suggest that the Ni/$Ti_3C_2T_2$ catalyst exhibits good catalytic activity and reasonable reaction kinetics towards electrochemical NRR. We believe that our study can provide a comprehensive understanding of the influence of the surface chemistry of MXenes on the catalytic performance of single metal atoms.

We appreciate the recommended literature from the reviewer. However, the suggested papers focus on the rational design of catalysts towards the nitrogen oxidation reaction [*ACS Nano* **2022**, *16*, 655] and the design of electrolyte in electrochemical NRR [*Adv. Energy Mater.* **2021**, *11*, 2101699], which will be of value for other studies. Therefore, we did not consider them relevant for the current work.

In the manuscript, we have added following discussion in the first section:

In addition, studies focusing on the catalytic performance of single atoms supported on MXenes with a mixture of termination groups are limited in number. Therefore, it is necessary to take the OH terminations into consideration for both the stabilization of single metal atoms and their influence on the catalytic performance.

2. When calculation the change of Gibbs free energy for each elementary step at zero potential, the changes of the zero-point energy are usually applied. However, in supporting information, I didn't find this part in calculation section.

Response: In this study, the contribution of the zero potential energy to the change of Gibbs free energy has been included in the vibrational enthalpy ($H^{vib}(T)$). For each elementary step of NRR, the reaction intermediates (N_xH_y) are chemisorbed to the transition metal atoms. The contribution to the Gibbs free energy from vibrational degrees of freedom is included by calculating the vibrational enthalpy $H^{vib}(T)$ and the vibrational entropy S^{vib}, as suggested by a previous study [*Chem. Mater.,* **2021**, *33*, 9108]. The vibrational enthalpy is defined by (eq. S3):

$$H^{vib}(T) = k_B \sum_i \left(\frac{h\nu_i}{2k_B} + \frac{h\nu_i}{k_B T} \frac{1}{e^{\frac{h\nu_i}{k_B T}} - 1} \right).$$

in which the first term is the zero-point energy (ZPE).

3. How about the effect of other termination group such as Cl, Br, I, S and Te on catalytic activity for SAC-supported MXene?

Response: We have performed supplementary calculations to elucidate the influence of other terminations on the catalytic performance of single Ni atom catalysts. Herein, we replaced OH terminations in $Ni/Ti_3C_2O_{0.19}(OH)_{1.81}$ with Cl, Br, I, and Te. The influence of termination groups on the catalytic performance is studied from three aspects as proposed in the manuscript: the adsorption of single Ni atom, the adsorption of N_2, and the adsorption of NH_3. As listed in the **Table S2**, the halogen terminations (F, Cl, Br, and I) exhibit limited promotions on the catalytic performance, in which the NH_3 binds strongly on the Ni atom. In addition, the S termination possesses similar performance as $Ti_3C_2O_2$. Interestingly, the Te terminations may boost the catalytic performance due to the reduced adsorption of NH_3. However, the synthesis of multi-layered S and/or Te terminated Ti_3C_2 MXenes requires high temperature and cannot be proceeded in traditional solvent [*Science,* **2020**, *369*, 979]. To the best of our knowledge, the fabrication of single sheets of $Ti_3C_2S_2$ and $Ti_3C_2Te_2$ has not been reported yet. Therefore, the application and the potential catalytic performance of such MXenes in SACs are beyond the scope of this study.

In the manuscript, the corresponding information has been added:

Similar results can be observed for other terminations including Cl, Br, I, and S. Interestingly, the $Ni/Ti_3C_2O_{0.19}Te_{1.81}$ exhibits positive adsorption energies for the 2nd NH_3, suggesting promising reaction kinetics for electrochemical NRR. However, the synthesis of multilayers of the Te terminated Ti_3C_2 requires high temperature (300°C to 600°C)[49] and the fabrication of the $Ti_3C_2Te_2$ monolayer has not been reported yet. Such obstacles would therefore hinder the further application of Te terminated Ti_3C_2 in single atom catalysis.

(49) Kamysbayev, V.; Filatov, A. S.; Hu, H.; Rui, X.; Lagunas, F.; Wang, D.; Klie, R. F.; Talapin, D. V. Covalent Surface Modifications and Superconductivity of Two-Dimensional Metal Carbide MXenes. *Science* **2020**, *369* (6506), 979–983.

Corresponding information has been added into the supporting information:

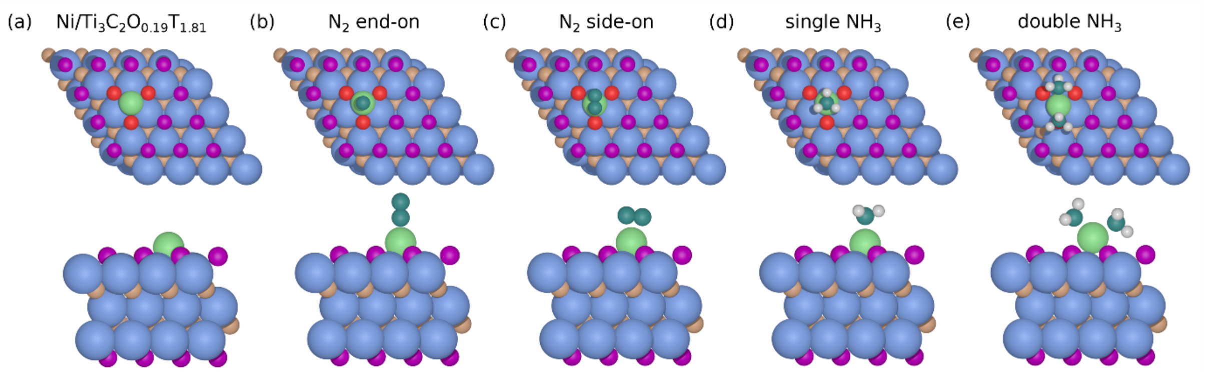

Figure S5. The optimized structures (top view for the upper panel and side view for the lower panel) for (a) Ni/$Ti_3C_2O_{0.19}T_{1.81}$ (T = F, Cl, Br, I, S, and Te), (b) the end-on adsorption of N_2, (c) the side-on adsorption of N_2, (d) single NH_3 adsorption, and (e) double NH_3 adsorption on the Ni/$Ti_3C_2O_{0.19}T_{1.81}$. The Ni, O, Ti, C, T, and N atoms are represented by the light green, red, blue, brown, purple, and dark green circles, respectively.

Table S2. The adsorption of Ni, N_2, and NH_3 on Ni/$Ti_3C_2T_2$ MXenes with different terminations in vacuum.

	Ni adsorption (eV)	N_2 adsorption (eV)		NH_3 adsorption (eV)	
		end on	side on	Single NH_3	2nd NH_3
Ni/$Ti_3C_2O_2$	1.57	-0.93	-0.44	-1.42	/
Ni/$Ti_3C_2O_{0.19}(OH)_{1.81}$	2.03	-1.39	-1.16	-0.45	0.15
Ni/$Ti_3C_2O_{0.19}F_{1.81}$	1.95	-0.99	-0.51	-1.21	-0.46
Ni/$Ti_3C_2O_{0.19}Cl_{1.81}$	1.92	-1.01	-0.64	-1.15	-1.08
Ni/$Ti_3C_2O_{0.19}Br_{1.81}$	1.95	-1.03	-0.55	-1.06	-1.00
Ni/$Ti_3C_2O_{0.19}I_{1.81}$	1.93	-1.02	-0.75	-0.96	/[a]
Ni/$Ti_3C_2O_{0.19}S_{1.81}$	1.52	-0.95	-0.74	-1.48	-1.96
Ni/$Ti_3C_2O_{0.19}Te_{1.81}$	1.84	-0.99	-0.86	-1.03	0.21

[a]No stable adsorption configuration

In order to understand the effects of other termination groups on the catalytic performance, the catalytic performance of single Ni atom supported on $Ti_3C_2O_{0.19}T_{1.81}$ (T = F, Cl, Br, I, S, and Te) MXenes are investigated based on proposed screening criteria. As listed in Table S2, Ni/$Ti_3C_2O_{0.19}S_{1.81}$ exhibits similar catalytic performance as the Ni/$Ti_3C_2O_2$, in which strong NH_3 adsorption would prohibit further catalytic cycles. In addition, halogen terminated Ti_3C_2 MXenes exhibit weaker Ni adsorption but can enhance the N_2 adsorption comparing to the O terminated Ti_3C_2. Nevertheless, the potential catalytic performance for Ni/$Ti_3C_2O_{0.19}X_{1.81}$ (X = F, Cl, Br, and I) is limited by the strong NH_3 adsorption. Interestingly, the Ni/$Ti_3C_2O_{0.19}Te_{1.81}$ exhibits positive adsorption energies for the 2nd NH_3 (0.21 eV), suggesting promising reaction kinetics for electrochemical NRR. However, practical challenges for multilayers of Te terminated Ti_3C_2 MXenes ($Ti_3C_2Te_2$) remain at the harsh synthetic conditions (300°C to 600°C in molten alkali metal halides).[14] Furthermore, the fabrication of the $Ti_3C_2Te_2$ monolayer has not been reported yet, which prevents further applications in single atom catalysis.

4. From Figure 4, it is clear that the O/OH terminations can not only change the energy of the N_2 hydrogenation at the first step, it also has an importance effect on other steps via different reaction pathway. Therefore, more discussion should be added to focus on each reaction step.

Response: We thank the reviewer for the valuable suggestion. We have added more discussion regarding to the reaction pathway and the energy profiles of the electrochemical NRR on Ni/$Ti_3C_2O_2$ and Ni/$Ti_3C_2T_2$.

The following discussion has been added into the manuscript:

Moreover, the second hydrogenation step along the enzymatic pathway exhibits an exothermic characteristic on the Ni/$Ti_3C_2T_2$, while a Gibbs free energy barrier of 0.14 eV should be overcome on the Ni/$Ti_3C_2O_2$ (Figure 4b). Such discrepancy in Gibbs free energy profiles indicates that the single Ni atom exhibits different selectivity on the $Ti_3C_2T_2$ and $Ti_3C_2O_2$. As a result, the enzymatic pathway is no longer energetically favored while the alternating pathway exhibits the lowest overall Gibbs free energy of 1.05 eV on the Ni/$Ti_3C_2O_2$. Despite, the catalytic performance of the Ni/$Ti_3C_2O_2$ catalyst is profoundly limited by the strong interactions between the single Ni atom and NH_3 molecules (G(NH_3) = -1.60 eV and G(N_2H_6) = -1.51 eV).

Reviewer 2:

Niu et al. present a systematic computational search for single-atom catalysts for nitrogen reduction supported on MXene substrates. Specifically, they compare adsorption energies of single metal atoms vs dimers as a proxy for obtaining well-dispersed catalyst atoms on the substrates, and those of nitrogen vs ammonia to identify candidates that strongly bind the reactant but can release the product. Based on these metrics, they identify Ni supported on the Ti3C2T2 MXene as the best candidate and map out the energetics of the nitrogen reduction reaction on this material in detail. Overall, the study is well-organized, systematically scanning the complex space of materials to find a promising catalyst, and is well-written, meriting publication in JPCL.

Response: We thank the reviewer for the many suggestions which helped us strengthening our manuscript.

It would strengthen the manuscript to further justify the choice of adsorption energies as a metric for the catalyst stability. In particular, all computed energies are for neutral adsorbates in vacuum. This could change substantially in the electrochemical environment, especially given the potentially harsh potentials required for nitrogen reduction.

Response: We have performed supplementary calculations to investigate the solvation effect on the stability and the catalytic performance of single transition metals supported on the $Ti_3C_2T_2$ MXenes. Herein, the implicit model implemented in VASPsol is employed to study the solvation effect as suggested by previous studies [*Appl. Surf. Sci.*, **2022**, *572*, 151417 and *Phys. Chem. Chem. Phys.*, **2021**, *23*, 4178]. Our calculations have shown that with the implicit solvation model, the adsorption of single transition metal atoms on the $Ti_3C_2O_2$ MXene is enhanced (**Figure 1**). Such enhanced adsorption of metal atoms indicates that the TM/$Ti_3C_2O_2$ catalysts exhibit higher stability with implicit solvation. Of importance, the stability of the single atom adsorption on the $Ti_3C_2O_2$ is further assessed by considering corrosion reactions of TM/$Ti_3C_2O_2$, in which the corrosion potentials for each transition metal atoms are calculated (**Table S1** and **eq S9-S14**). As a result, most transition metals can be stablized in the form of single atoms on the $Ti_3C_2O_2$ when the applied potential is smaller than -1 V vs. SHE. Considering that the electrochemical NRR takes place at the cathode and the negative potentials are required, the single atom dispersion of transition metal atoms can thus be guaranteed (detailed discussion in the response to reviewer 3 below).

Despite, the ability for single atoms to bind the N_2 molecule is decreased based on the implicit solvation model. As a result, the N_2 will not be stablized on the transition metal atoms such as Sc, Ti, Zr, Nb, and Mo supported on the $Ti_3C_2O_2$ (**Figure S2**). However, limited suppression on the NH_3 adsorption has been observed with implicit solvation model (**Figure S3**), in which the NH_3 maintains stronger interactions with single transition metal atoms than that of N_2 in solution. In addition, further calculations have revealed that introducing OH terminations to the Ti_3C_2 MXene is still an effective approach to promote the catalytic performance in solution. The updated adsorption energies for the N_2 and NH_3 on TM/$Ti_3C_2O_2$ and TM/$Ti_3C_2T_2$ are summarized in **Figure 2**. As shown in **Figure 2b**, the adsorption energy of the NH_3 on the TM/$Ti_3C_2T_2$ MXene is significantly decreased. For metal atoms including Fe, Co, V, and Ni, the adsorption of NH_3 is energetically less favored by more than 1 eV. Specifically, the NH_3 exhibits positive adsorption energies on Ag, Nb, and Mo catalysts, indicating a spontaneous desorption. Furthermore, the capability of single atoms to capture N_2 is strengthened on the $Ti_3C_2T_2$ support. As shown in **Figure 2c-2d**, the N_2 tends to bind stronger on single atoms supported on $Ti_3C_2T_2$. For example, the N_2 exhibits positive adsorption energies on Nb, Mo, Ti, and Zr atoms supported on the $Ti_3C_2O_2$, while such adsorption is significantly promoted when metal atoms are anchored on the $Ti_3C_2T_2$. Despite that Nb and Mo supported on the $Ti_3C_2T_2$ possess ideal N_2 and NH_3 adsorption, Nb and Mo SACs may exhibit unstable catalytic performance as the N_2 adsorption on Nb and Mo is highly sensitive to OH terminations. Furthermore, the co-adsorption of two NH_3 molecules on single atoms with implicit solvation model is investigated. As shown in **Figure 3**, the 2nd NH_3 exhibits stronger than that of the 1st NH_3 under solution condition for most of single atoms. Consequently, the capture of the N_2 and further hydrogenation steps would be hindered. Of importance, only Ni exhibits positive adsorption towards the 2nd NH_3 molecule, suggesting that the single Ni atom can exhibit fast kinetics, which is beneficial to further reduction reactions. To summarize, the solvent effect can enhance the single atom adsorption of TM on the $Ti_3C_2O_2$ and decrease the adsorption of NH_3 on the TM/$Ti_3C_2T_2$, which would promote the catalytic performance. Finally, the solvation effect on the Gibbs free energy profile of NRR on Ni/$Ti_3C_2T_2$ has been clarified. As shown in **Figure S4**, both the highest relative Gibbs free energy and the limiting free energy barrier have been reduced after including the solvation effect. To be specific, the limiting barrier decreases from 1.56 eV (vacuum) to 1.36 eV (implicit solvation), indicating a promoted catalytic activity of Ni/$Ti_3C_2T_2$. Besides, the relative Gibbs free energy of NH_3+NH_3 species (N_2H_6 in **Figure S4**) increases to -0.04 eV, which would accelerate reaction kinetics.

We have added more discussion and updated figures in the manuscript:

Herein, the adsorption behavior of transition metal atoms is investigated under both vacuum and implicit solvent conditions.

Moreover, single transition metals tend to bind stronger in the solvent. As seen in Figure 1b, most transition metals (except Ru, Rh, and Pd) exhibit stronger single atom adsorption energies with implicit solvent model. Consequently, the possibility to achieve single-atom dispersion is enhanced with implicit solvation.

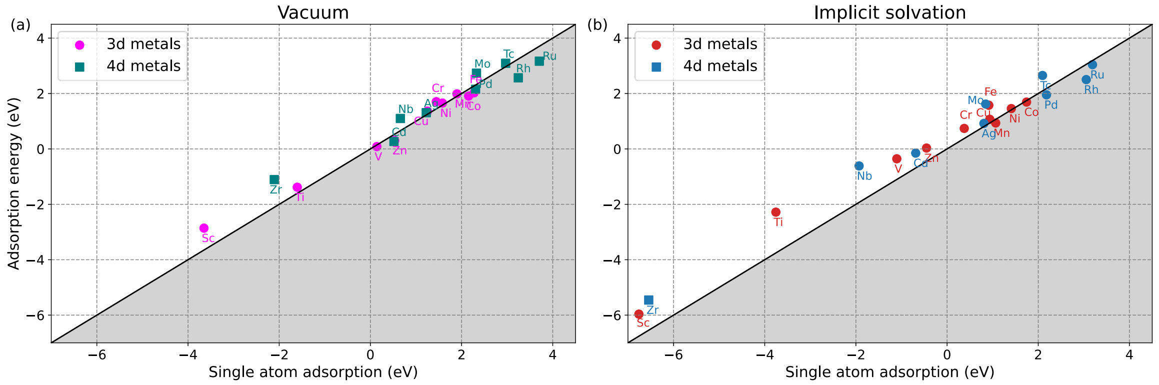

Figure 1. The adsorption energies of single transition metals and metal dimers on the $Ti_3C_2O_2$ MXene in (a) vacuum and (b) implicit solvation, calculated with eq. (1).

As seen in Figure S2c, N_2 exhibits positive adsorption energies on transition metals with less valence electrons (Sc, Ti, Zr, Nb, and Mo), suggesting limited catalytic activity. Nevertheless, for Co, Ni, Cu, Rh, and Pd, the N_2 can be stabilized in both end-on and side-on configurations ($E_{ad}^{N_2}$ < -0.2 eV).

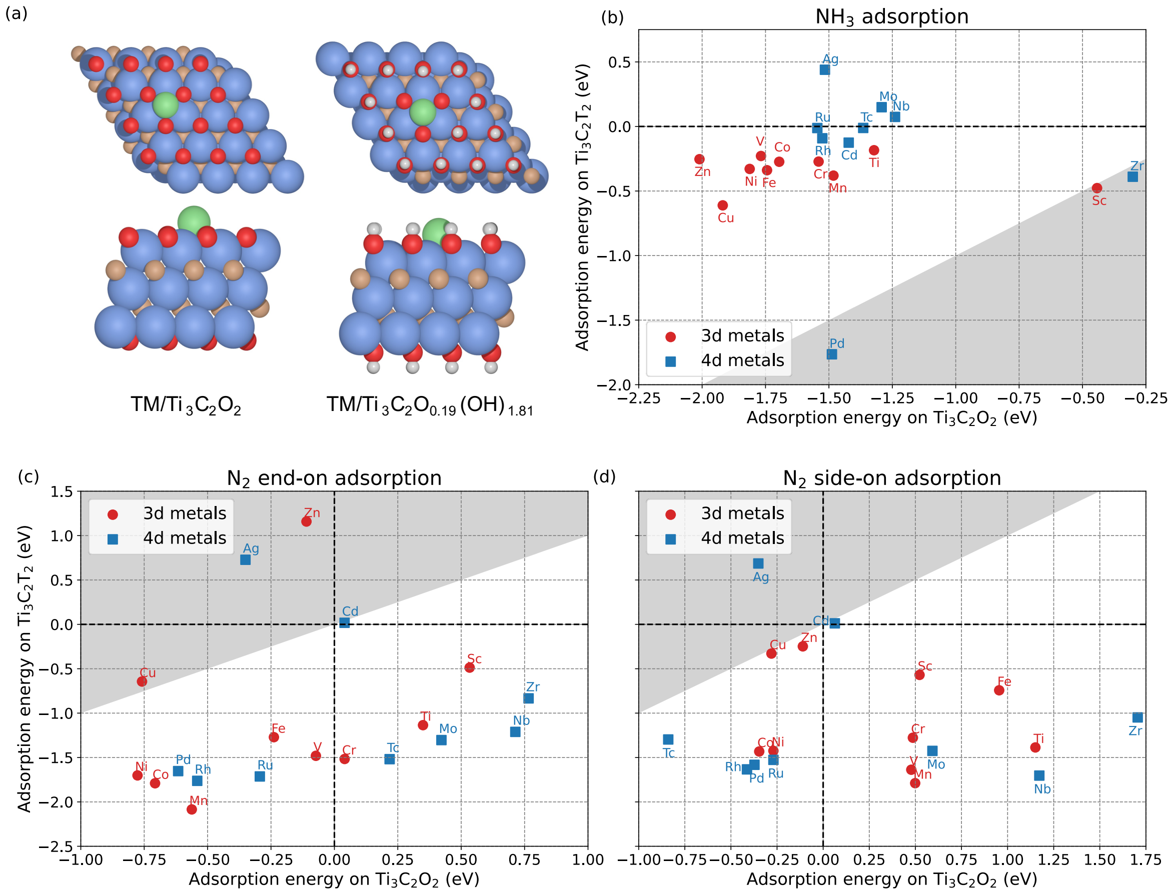

Figure 2. (a) The optimized structures for the TM/$Ti_3C_2O_2$ and TM/$Ti_3C_2O_{0.19}(OH)_{1.81}$ ($Ti_3C_2T_2$ for simplification). (b) The adsorption energies of the NH_3 on the TM/$Ti_3C_2O_2$ and TM/$Ti_3C_2T_2$. The adsorption energies of N_2 on the TM/$Ti_3C_2O_2$ and TM/$Ti_3C_2T_2$ in (c) end-on configuration and (d) side-on configuration. The implicit solvation model is employed for all adsorption energies. The Ti, C, O, H, and TM atoms in (a) are represented by the blue, brown, red, white, and green spheres, respectively.

Notably, the NH_3 possesses positive adsorption energies on Ag, Nb, and Mo single atoms, indicating a spontaneous desorption of NH_3.

As seen in Figure 2c and 2d, the adsorption N_2 is significantly strengthened on Mo, Nb, Cr, and Ti supported on the $Ti_3C_2T_2$, while N_2 exhibits positive adsorption energy on the same single atoms supported on the $Ti_3C_2O_2$.

To this end, single atoms (Fe, Co, Ni, V, Nb, and Mo) supported on the $Ti_3C_2T_2$ MXene can be considered as the promising catalysts towards electrochemical NRR due to the desired adsorption behavior of both N_2 and NH_3. However, the capability to bind N_2 for V, Mo, and Nb single atoms is highly sensitive to the amount of OH terminations on the MXene, resulting in unstable catalytic performance.

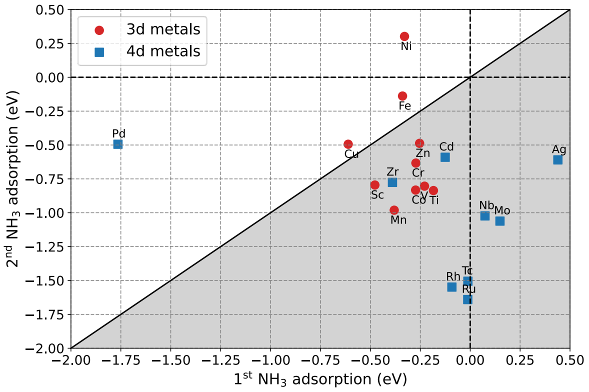

Figure 3. The comparison of the adsorption energy of the 1st NH_3 and the 2nd NH_3 molecule on the $TM/Ti_3C_2T_2$ MXene with implicit solvation model.

The adsorption energies of N_2 and NH_3 on the $TM/Ti_3C_2O_2$ are updated in supporting information:

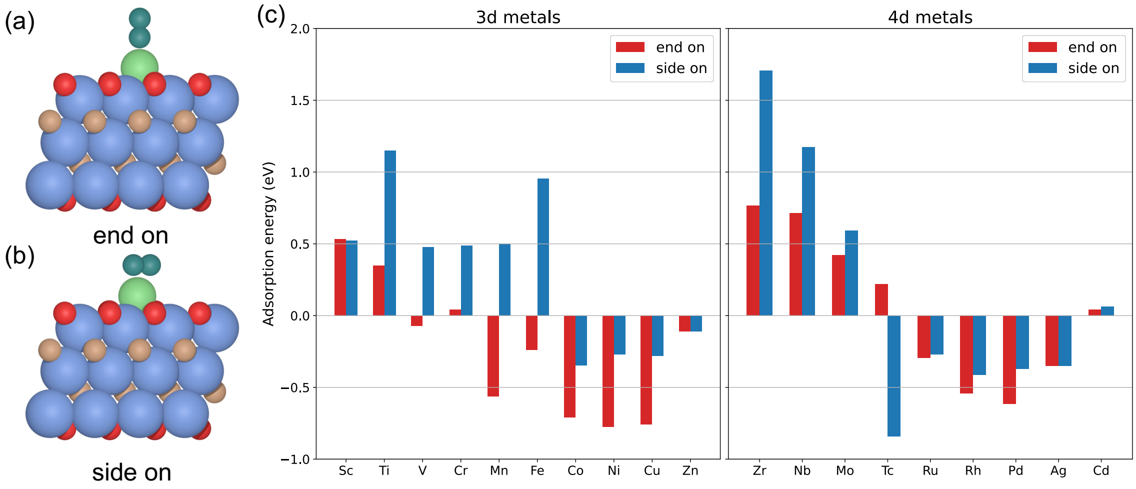

Figure S2. Optimized structures for N_2 adsorption on the SAC in (a) end-on configuration and (b) side-on configuration. (c) The adsorption energies of the N_2 on SACs with implicit solvation model. Ti, C, O, N, and TMs in (a) and (b) are represented by the blue, brown, red, dark green, and light green circles, respectively.

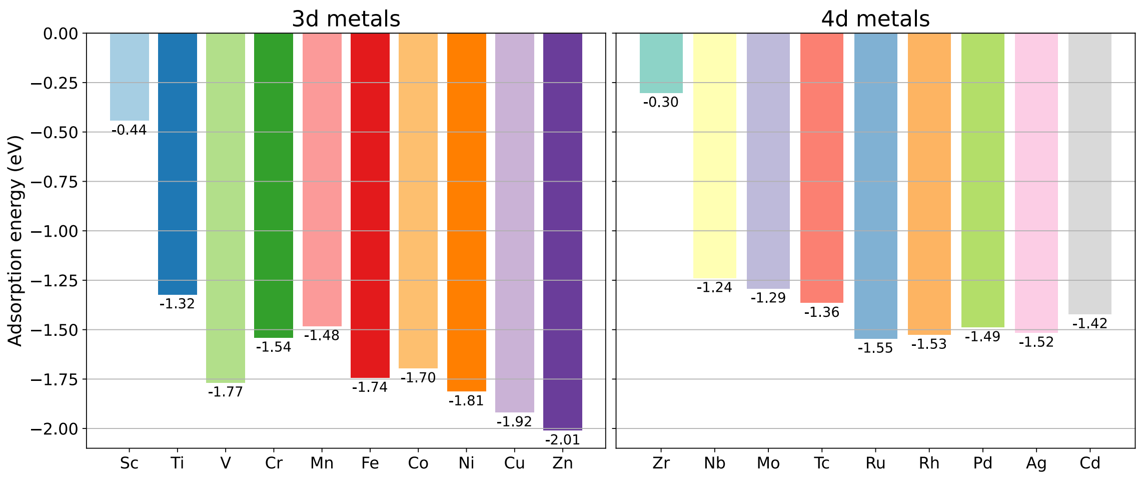

Figure S3. The adsorption energy of NH_3 on the TM/$Ti_3C_2O_2$ MXenes with implicit solvation model.

In the manuscript, following information has been added:

Furthermore, our calculations show that the Ni/$Ti_3C_2T_2$ exhibits better catalytic activity under implicit solvation condition. As seen in Figure S4, the limiting step for the Ni/$Ti_3C_2T_2$ along the enzymatic pathway is decreased from 1.56 eV to 1.36 eV. In addition, the relative Gibbs free energy of *NHNH is reduced to -0.11 eV in solution, indicating a more stable intermediate. Of importance, the accelerated reaction kinetics can be expected in the solution because the relative Gibbs free energy of N_2H_6 has been increased from -0.28 eV (in vacuum) to -0.04 eV (in implicit solvation).

In supporting information, we have added the following section:

The Gibbs free energy profile for enzymatic pathway.

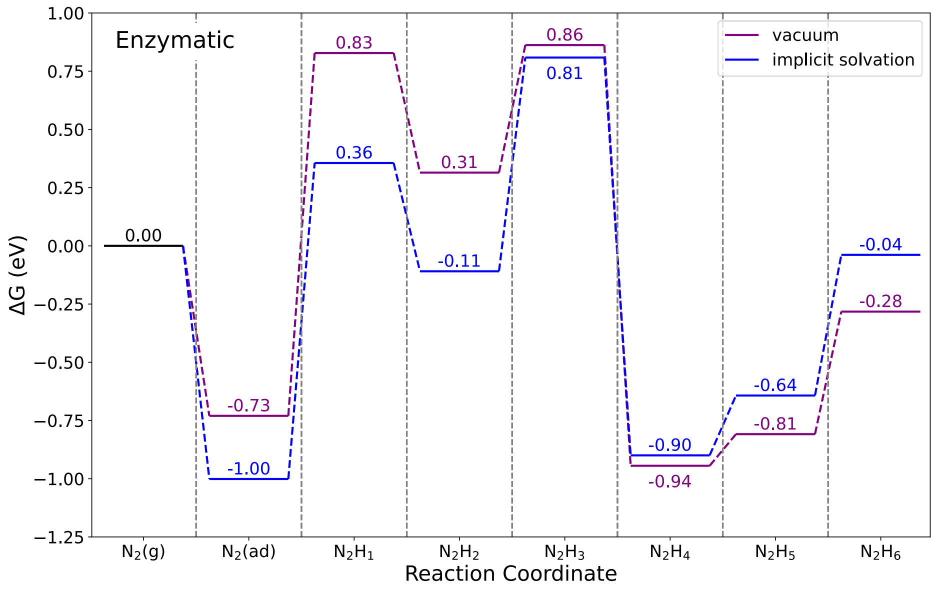

Figure S4. The Gibbs free energy profile for the electrochemical NRR on Ni/$Ti_3C_2T_2$ along the enzymatic pathway in vacuum (purple) and with implicit solvation (blue).

Figure S4 shows the influence of the implicit solvation effect on the catalytic performance of Ni active sites. As seen, the N_2 adsorption is more stable in the solution than in vacuum. The limiting Gibbs free energy barrier has been decreased from 1.56 eV to 1.36 eV, indicating the catalytic performance of the Ni atom is promoted in the solvent condition. In addition, reaction intermediate states such as *NNH and *NHNH bind stronger to the Ni in the solution, leading to a promotion of reaction kinetics. Furthermore, the implicit solvation effect exhibits positive effect on the adsorption of NH_3, in which the Gibbs free energy of co-adsorption of two NH_3 molecules (N_2H_6) is decreased from -0.28 eV to -0.04 eV. Such weak adsorption of NH_3 indicates the Ni active sites can be available rapidly, resulting in accelerated reaction kinetics.

Similarly, the reaction analysis is based on proton-coupled electron transfer steps, which could be a poor approximation for several of the nitrogen reduction intermediates. Qualifying the results shown in the manuscript to indicate these potentially important effects would be useful.

Response: The Gibbs free energy profile for electrochemical NRR is calculated based on the computational hydrogen electrode (CHE) method proposed by Nørskov [*J. Phys. Chem. B,* **2004**, *108(46)*, 17886], in which the chemical potential of the H^+/e^- pair is equal to half of the Gibbs free energy of H_2 (pH = 0, p = 1 bar, T = 298.15 K). Such method has been widely employed for theoretical calculations of various electrochemical reactions including oxygen reduction reactions [*Nat. Catal.*, **2021**, *4*, 463], CO_2 reduction reactions [*Nat. Catal.*, **2021**, *4*, 1024], and nitrogen reduction reactions [*J. Mater. Chem. A*, **2021**, *9*, 15217]. In the present work, we focus on the cathode reaction of electrochemical NRR at ambient condition and pH = 0. The reaction at cathode is:

$$N_2(g) + 6H^+ + 6e^- = 2NH_3$$

in which 6 proton-coupled electron transfer steps are involved.

Corresponding information has been added into the Supporting information:

The computational hydrogen electrode (CHE) method proposed by Nørskov et al is employed to calculate Gibbs free energy profiles for electrochemical NRR,[5] in which the electrochemical nitrogen reduction reaction is: $N_2(g) + 6H^+ + 6e^- = 2NH_3$, including 6 proton- electron pair ($H^+ + e^-$) transfer steps.

According to the CHE model, the chemical potential of the H^+/e^- pair under standard conditions is equal to the half of the Gibbs free energy of the H_2 (pH = 0, *p* = 1 bar, T = 298.15 K). The Gibbs free energy of H_2 is defined by:

$$G_{H_2}(p,T) = H_{H_2}^{elec} + H_{H_2}^{trans}(T) + H_{H_2}^{rot}(T) + H_{H_2}^{vib}(T) - TS_{H_2}(p,T), \quad \text{(S5)}$$

where $H_{H_2}^{elec}$, $H_{H_2}^{trans}(T)$, $H_{H_2}^{rot}(T)$, and $H_{H_2}^{vib}(T)$ are the electronic, translational, vibrational, and rotational enthalpies of H_2, respectively. The translational enthalpy is defined by:

$$H_{H_2}^{trans}(T) = \frac{3}{2}k_B T, \quad (S6)$$

the rotational enthalpy for H_2 is defined by:

$$H_{H_2}^{rot}(T) = \frac{3}{2}k_B T, \quad (S7)$$

the vibrational enthalpy and entropy are defined the same as eq. S3. The tabulated value of entropy of the H_2 (S_{H_2}) from as used.[7]

(7) Chase, M. W. NIST-JANAF Thermochemical Tables. *J. Phys. Chem. Ref. Data* **1998**, *9*, 1310.

Reviewer 3:

The authors investigated the potential of a single transition metal atom anchored on the $Ti_3C_2T_2$ (T = O and/or OH) MXene as electrocatalyst for NRR and the effect OH terminations on the catalytic performance. While a key point is that the adsorption energy of single transition metal Ni on the $Ti_3C_2O_2$ MXene is positive, see fig.1 and formula (1), that means Ni is not adsorbed on the surface of $Ti_3C_2O_2$, based on this positive adsorption energy, we do not have any next step for NRR or other electrocatalysis performances on Ni@ $Ti_3C_2O_2$ system. This paper is not recommended because it does not provide correct physical insights.

Response: We thank the reviewer for the comment.

In our work, the adsorption energy of metal atoms is employed as a descriptor to characterize the possibility of agglomeration, in which metals in the gray region in **Figure 1** may form cluster on the MXenes.

For an electrochemical reaction, it is necessary to evaluate the stability of single atom catalysts in solution. Herein, we performed the single atom adsorption on the $Ti_3C_2O_2$ with implicit solvent model and calculated required potential for stabilizing single atoms. To demonstrate the stability of single metal atoms, we have considered the corrosion of single metal atoms. Corresponding reactions are defined by:

$$M(ad) \rightarrow M^{n+} + ne^-$$

in which M refers to the metal atom, and *n* refers to the number of electrons the cation loses after dissolution. Specifically, for the Nb and Mo, the corrosion reactions are defined as:

$$Nb(ad) + 3H_2O \rightarrow HNbO_3 + 5H^+(aq) + 5e^-$$

and

$$Mo(ad) + 4H_2O \rightarrow HMoO_4^- + 7H^+(aq) + 6e^-,$$

because the most stable ions for Nb and Mo are $HNbO_3$ and $HMoO_4^-$, respectively. At pH = 0, the corrosion potential (U) for metal atoms is defined as:

$$U(M) = -\frac{E_{ad}(M) - E_{formation}(m^{n+})}{ne},$$

$$U(Nb) = -\frac{E_{ad}(Nb) - E_{formation}(HNbO_3) + 3E(H_2O)}{5e},$$

and

$$U(Mo) = -\frac{E_{ad}(Mo) - E_{formation}(HMoO_4^-) + 4E(H_2O)}{7e}.$$

The corrosion potentials refer to the maximum potential for transition metals to maintain their single-atom adsorption. As listed in **Table S1**, the corrosion potentials for most 3d and 4d metals are in the range from -1.0 to 0.5 V. In addition, the electrochemical NRR not only take places at the cathode

where the negative potential is applied but also requires negative potential to procced hydrogenation steps. Specifically, the corrosion potential for Ni supported on $Ti_3C_2O_2$ in solution is -0.941 V, which is larger than the potential to proceed the NRR on Ni active site (-1.36 eV, seen from **Figure S4**). Therefore, it is reasonable to deduce that transition metal atoms will maintain the single atom dispersion in the electrochemical environment for nitrogen reduction reactions. In addition, the effect of the implicit solvation model on the adsorption of the single metal atoms, N_2, and NH_3 has been elaborated in the response to the review 2 (**Figure 1-3, Figure S1-S4**), in which the single Ni atom can be stablized on the $Ti_3C_2T_2$ MXene and exhibits good catalytic performance towards electrochemical NRR.

In the manuscript, we have added information as follows:

In addition, the stability for the single atom adsorption is assessed by the corrosion potential of $TM/Ti_3C_2O_2$ in solution environment. The corrosion reactions of transition metals are the dissolution of transition metals to form the most stable cations/anions (eq. S9-S11) and corrosion potentials (calculated by eq. S12-S14) at pH = 0 are the highest potentials to stabilize the single atom adsorption (Table S1). It is found that corrosion potentials for the majority of single transition metal atoms on the $Ti_3C_2O_2$ are in the range from -1.0 V to 0.2 V (vs. SHE). Taking into account that the electrochemical NRR requires negative potentials, the corrosion can be effectively prevented during the NRR process.

Corresponding section has been added into the supporting information:

Table S1. The adsorption energies and the potential requirement for stabilizing the single atoms.

Metal	Adsorption energy (eV)	Corrosion potential U (V vs. SHE at pH=0)	Reference ion
Sc	-6.760	0.227	Sc^{3+}
Ti	-3.755	0.043	Ti^{3+}
V	-1.103	-0.576	V^{2+}
Cr	0.377	-1.043	Cr^{2+}
Mn	1.066	-1.715	Mn^{2+}
Fe	0.916	-0.867	Fe^{2+}
Co	1.743	-1.154	Co^{2+}
Ni	1.409	-0.941	Ni^{2+}
Cu	0.936	-0.418	Cu^{+}
Zn	-0.451	-0.537	Zn^{2+}
Zr	-6.545	0.191	Zr^{4+}
Nb	-1.930	-0.193	$HNbO_3$
Mo	0.848	0.006	$HMoO_4^-$
Tc	2.093	/	/
Ru	3.190	-0.816	Ru^{2+}
Rh	3.053	-0.926	Rh^{2+}
Pd	2.181	-0.176	Pd^{2+}
Ag	0.808	-0.009	Ag^{+}
Cd	-0.688	-0.058	Cd^{2+}

The corrosion potential of single transition metals on the $Ti_3C_2O_2$ in the electrochemical environment (pH = 0) has been evaluated according to dissolution reactions, which are defined as:
for metal cations:

$$M(ad) \rightarrow M^{n+} + ne^{-}, \tag{S9}$$

for Nb, the $HNbO_3$ is the most stable form:

$$Nb(ad) + 3H_2O \rightarrow HNbO_3 + 5H^{+}(aq) + 5e^{-}, \tag{S10}$$

and for Mo, the $HMoO_4^{-}$ anion is the most stable:

$$Mo(ad) + 4H_2O \rightarrow HMoO_4^{-} + 7H^{+}(aq) + 6e^{-}. \tag{S11}$$

Correspondingly, the corrosion potential for transition metals is defined as:

$$U(M) = -\frac{E_{ad}(M) - E_{formation}(m^{n+})}{ne}, \tag{S12}$$

$$U(Nb) = -\frac{E_{ad}(Nb) - E_{formation}(HNbO_3) + 3E(H_2O)}{5e}, \tag{S13}$$

and

$$U(Mo) = -\frac{E_{ad}(Mo) - E_{formation}(HMoO_4^{-}) + 4E(H_2O)]}{7e}. \tag{S14}$$

in which $E_{ad}(M)$, $E_{formation}(m^{n+})$ and *n* refer to the adsorption energy of single M atom on the $Ti_3C_2O_2$ in solution (the free energy for solid phases are estimated by the electronic enthalpy at 0 K), the formation energy of metal cations obtained from the dissolution potential of pure metals in experiment as tabulated in Ref. [12] and Ref. [13], and the number of positive charges of the cation, respectively. Note that the formation free energy of the H^{+} ions are not included in the corrosion potentials since it is zero by definition for pH = 0.

(12) Wagman, D. D.; Evans, W. H.; Parker, V. B.; Schumm, R. H.; Halow, I.; Bailey, S. M.; Churney, L.; Nuttall, R. L. The NBS Tables of Chemical Thermodynamic Properties: Selected Values for Inorganic and C_1 and C_2 Organic Substances in SI Unit. *J. Phys. Chem. Ref. Data* **1982.**
(13) Pourbaix, M. *Atlas of Electrochemical Equilibria in Aqueous Solutions*; National Association of Corrosion Engineers: Houston, Texas, 1974.
